# OPC3/LEW: A New Flexible Three-Site Water Model to Simulate Low-Electrostatic Environments

**DOI:** 10.1021/acsomega.6c08432

**Published:** 2026-10-21

**Authors:** José Raúl Romero-Figueroa, Jorge Alberto Aguilar-Pineda, Minerva González-Melchor

**Affiliations:** Instituto de Física “Luis Rivera Terrazas”, 211699 Benemérita Universidad Autónoma de Puebla, Av. San Claudio, Cd. Universitaria, Apdo. Postal J-48, 72570 Puebla, México

## Abstract

One of the main goals of computational simulations is to closely reproduce the environmental conditions in which biomolecules perform their physiological functions. However, the choice of water model is often based more on computational cost than on its effect on the molecular environment, as is the case with membrane proteins. These systems fulfill their biological function in low-polarity molecular environments that the most common or currently available water models do not reproduce accurately. In this work, we present a new flexible water model with three interaction sites that accurately represents the low-polarity electrostatic environment typically observed in the proximity of membrane systems. Using molecular dynamics simulations and based on the interaction parameters of OPC3, this new low-electrostatic water model (OPC3/LEW) reproduces dielectric constants close to 20. Following the methodology of Aguilar-Pineda et al. for developing low-electrostatic models by opening the H–O–H angle, the bulk density and surface tension of blood plasma under physiological conditions were selected as target properties. The performance of the OPC3/LEW model was validated by evaluating nine thermodynamic properties as a pure component and its ability to preserve helical structures as a solvating medium for eight amphipathic peptides. Outcomes were compared with those obtained using four water models with three interaction sites: OPC3, OPC3-B, SPC/E, and TIP3P. Findings show that the OPC3/LEW model reproduces several properties of bulk water more accurately than models such as SPC/E and TIP3P at the evaluated temperatures. However, its greatest strength lies in preserving the helical structures of peptides that otherwise would adopt disordered states. Although its main limitation is the geometry of its open structure, which makes the model somewhat unrealistic, the findings suggest that the OPC3/LEW model could be used in systems involving low-polarity environments, such as membrane proteins.

## Graphical abstract

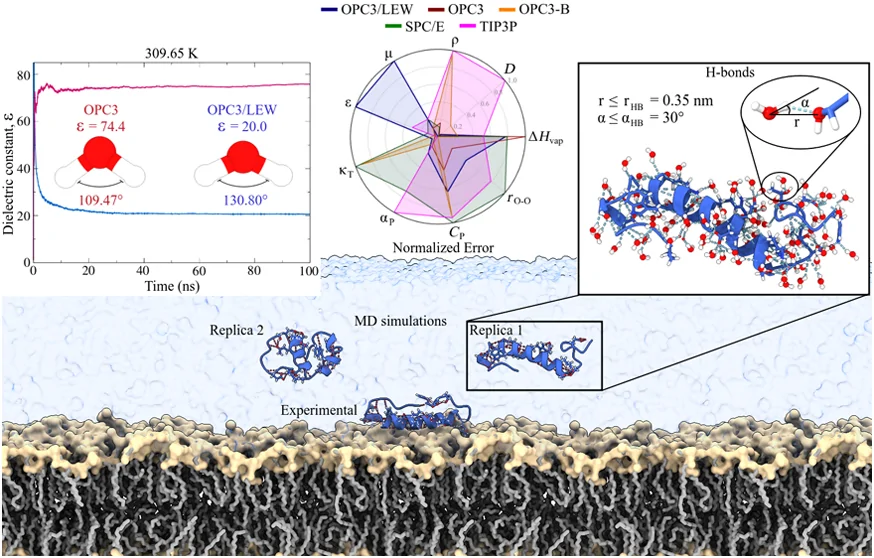

## Introduction

α-helical membrane proteins (αHMPs) are currently a major therapeutic target, with approximately 60% of all approved drugs targeting them.^1–3^ Expressed by nearly 30% of genes in most organisms, including humans, they participate in a wide range of physiological mechanisms, and their dysfunction is associated with a diverse array of diseases.^4^ Located in both the plasma membrane and endomembranes, the αHMPs diversity includes G protein-coupled receptors (GPCRs), ion channels, transporters, and cell receptors, among others.^1^ Despite their relevance to human health, the experimental study of αHMPs remains a significant challenge due to the complexity of ensuring optimal conditions for their extraction, purification, and preservation of their native structure.^2,5^ To address this problem, advanced scanning techniques have been developed, such as X-ray crystallography,^6,7^ nuclear magnetic resonance (NMR),^8,9^ and high-resolution cryogenic electron microscopy (cryo-EM), which can resolve biomolecular structures at quasi-atomic resolution.^5,10,11^ However, one of the biggest challenges for their analysis is the electrostatic environment surrounding αHMPs. Since the three-dimensional structure of biomolecules depends heavily on their environment, factors such as pH, pressure, temperature, and solvent properties are fundamental to describing their native structure.^12–14^ In the case of αHMPs, the native environment is extremely complex, as it interacts with lipid molecules, other membrane proteins, water, and various biomolecules, influencing their structure and, therefore, their biological function.^2,15^

In this membrane environment, water behaves differently from the bulk aqueous phase. Experimental and theoretical studies have shown that complex interactions with lipid molecules alter the dynamic properties of water.^16–21^ One of these is the dielectric constant, which decays significantly in the vicinity of the cell membrane (<2.5 nm) to values that can range between 4 and 20.^22–24^ This low polarity differs from that of the plasma protein environment, which is close to that of water under physiological conditions (ϵ ≈ 74). The low-electrostatic environment plays a determining role in the accurate molecular description of membrane systems, as it directly affects the structural properties and the energy of electrostatic interactions, which is inversely dependent on the dielectric constant of the solvent.^25^ Cell recognition, protein-drug interactions, signal transduction, and oligomerization are just some examples of processes that can be affected by a local decrease in the dielectric environment.^14^

In this regard, numerous efforts have been made to study αHMPs once removed from their native environment. One of the most common methods is to use alcohols or mixtures of alcohols with water, such as 2,2,2-trifluoroethanol (TFE), to decrease the polarity of the medium.^14,24,26^ Another technique is the addition of lipid molecules to aqueous solutions, allowing them to interact with proteins while preserving protein structure.^5,14^ Notably, these studies have also observed that disordered peptides and proteins exhibit a dramatic increase in helical character due to the low polarity of the medium,^24,27–31^ thereby inducing the formation of amphipathic helices.^26^ This structural change is one of the steps in the process of penetration or pore formation in the cell membrane.^31,32^

Faced with this challenge, molecular simulations have become indispensable tools for addressing such issues.^1,33^ In particular, molecular dynamics (MD) has emerged as one of the most widely used approaches for analyzing macromolecular systems.^34^ MD is used to study processes occurring in cell membranes, including protein–membrane interactions,^34–36^ αHMPs oligomerization,^37,38^ self-organization of lipid bilayers,^39^ and lateral diffusion of receptors,^40^ among others. However, in molecular simulations, it is common to study these systems using water models developed to describe the environment of soluble proteins. Therefore, their success depends heavily on accurately capturing the complex solvation effects and the computational performance of the water model employed, since the solvent often constitutes a significant and integral part of the system.^41,42^

Due to their efficiency and low computational cost, the most popular solvent representations are the rigid, nonpolarizable, three-site models such as SPC/E^43^ and TIP3P.^44^ Recently, new models have been developed prioritizing the electrostatic properties of water (OPC3 ^45^) or the structural properties of biomolecules (OPC3-B^46^). Unfortunately, while these models have been widely used to reproduce certain properties of bulk water,^47–50^ they fail to capture the reduced-polarity environments observed experimentally in the vicinity of polar membranes.^51,52^ At membrane interfaces, rigid, nonpolarizable three-site water models pose difficulties primarily because their fixed charges cannot adapt to the highly heterogeneous electrostatic environment of a membrane, where dielectric properties vary drastically among bulk water, lipid head groups, and the hydrophobic core.

In recent work, Aguilar-Pineda et al. have addressed this topic and its importance in accurately describing the electrostatic environment in membrane systems using MD simulations. First, they analyzed the influence of the water model on the structural and energetic properties of the GPR40 receptor, using rigid (SPC/E and TIP4P^44^) and flexible (FBA/ϵ^53^ and TIP4P/ϵ_flex_ ^54^) water models.^52^ The results showed that the flexible models performed best (FBA/ϵ) and worst (TIP4P/ϵ_flex_) on the analyzed properties. Based on later models, they developed two flexible models that reproduced low-polarity environments and analyzed their effects on five peptides, the GPR40, and the Rv2617c proteins. Their findings showed that these new models promoted the formation of intramolecular hydrogen bonds, which helped preserve their tertiary structures more effectively.^30^ However, these models failed to improve the preservation of helical structures in the analyzed peptides compared to the original models.

The importance of the electrostatic environment of αHMPs in molecular simulations lies in the fact that these studies often represent the initial stages in various experimental investigations with biomolecules. Molecular recognition mechanisms, catalytic interactions, and drug development are just some of the processes that can be seriously affected by an inadequate representation of the molecular environment. For these reasons, we present the novel OPC3/LEW model in this work, a water force field designed to produce low-electrostatic environments in molecular simulations (targeting a dielectric constant ϵ ≈ 20). Its development was based on the interaction parameters of the OPC3 model and the methodology proposed by Aguilar-Pineda et al. for obtaining low dielectric constants in water models. Furthermore, two additional target properties were defined for reproduction: the bulk density of water and the surface tension of blood plasma at physiological temperature. Incorporating these properties significantly improves its performance as a solvating medium for helical peptide structures compared with other low-electrostatic models (FBA_mem_ and T4F_mem_). Validating its performance against other rigid, three-site water models (OPC3, OPC3-B, SPC/E, and TIP3P), greater helix conservation and promotion of intramolecular hydrogen bonds are observed. Furthermore, as a pure component, OPC3/LEW can reproduce physicochemical properties with lower error than popular models such as SPC/E and TIP3P. It is expected that this new model will accurately represent the complex molecular interactions at the membrane interface and, above all, can be used in more complex systems, such as those formed by membrane proteins in the lipid matrix.

## Computational Details

### Reparameterization Procedure of the Water Model

The novel structural and interaction parameters of the OPC3/LEW model were obtained from molecular dynamics (MD) simulations using the GROMACS 2021 suite and the OPLS/AA force field (FF).^55–58^ Taking the OPC3 parameters as initial values (Table 1) and following the Aguilar-Pineda et al. methodology,^30^ a scan of the H–O–H angle was performed, opening it until the total dipole moment of the system decreased, yielding a dielectric constant of approximately 20 (Figure 1a,b). According to the functional form of the OPLS/AA FF, the nonbonded interactions, modeled by Coulomb and Lennard-Jones (LJ) contributions, between a pair of atoms *i* and *j* can be described by a pairwise-additive potential

1 $$U\left(r_{\mathrm{ij}}\right)=\sum_{i}\sum_{j>i}\left\{\frac{q_{i}q_{j}}{4\pi \epsilon_{0}r_{\mathrm{ij}}}+\left[4\epsilon_{\mathrm{ij}}{\left(\frac{\sigma_{\mathrm{ij}}}{r_{\mathrm{ij}}}\right)}^{12}-{\left(\frac{\sigma_{\mathrm{ij}}}{r_{\mathrm{ij}}}\right)}^{6}\right]\right\}f_{\mathrm{ij}}$$

where, *r*_**i*j*_ represents the distance between the interacting atoms *i* and *j*, *q*_*i*_ and *q*_*j*_ are the partial charges on *i* and *j* atoms, ϵ_0_ is the permittivity of vacuum, σ_*ij*_ is the van der Waals diameter, and ϵ_*ij*_ is the well-depth for a pair of interacting atoms. The geometric mixing rules for cross interactions are σ_*ij*_ = (σ_*ij*_ σ_*ij*_)^0.5^ and ϵ_*ij*_ = (ϵ_*ij*_ ϵ_*ij*_)^0.5^. The scaling factor *f*_*ij*_ = 1.0, except for intramolecular interactions between atom pairs separated by three bonds, where the value is 0.5.

**Figure 1:**
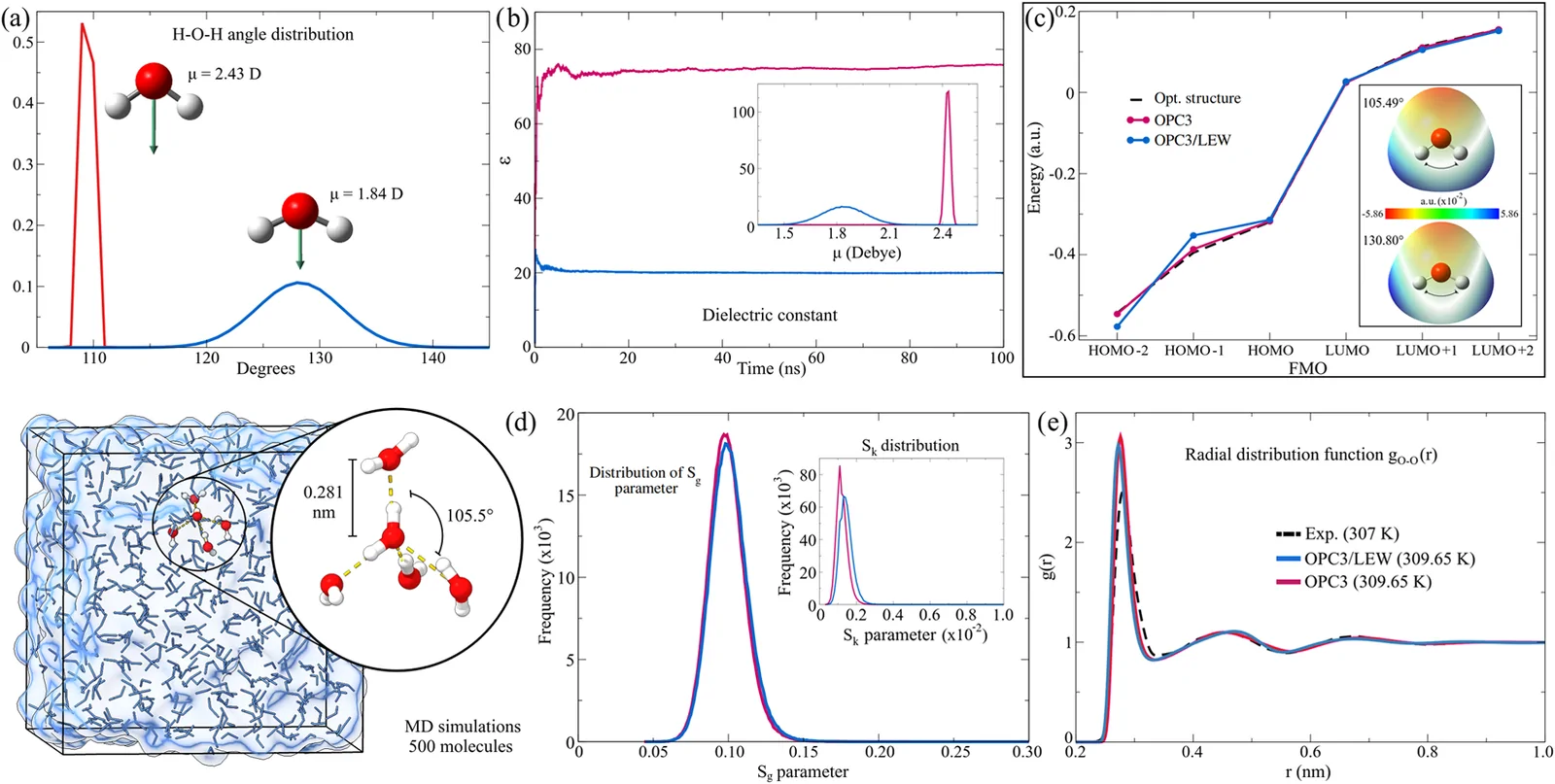
Process for finding the optimal angle at which water can reproduce a low-electrostatic environment. (a) Scan of the HOH angle to decrease the dipole moment of the OPC3 water. (b) Dielectric constants obtained in MD simulations for OPC3 and OPC3/LEW models at 298.15 and 309.65 K. (c) Energy profiles of frontier molecular orbitals of the water molecule using the structural parameters of OPC3 and OPC3/LEW models. DFT calculations were performed in the B3LYP//6–31 + G­(d) level of theory. The inset shows the electrostatic potential surfaces for both models. (d) Tetrahedral analysis for the OPC3/LEW configurations. (e) Radial distribution function comparisons for original and new model at 298.15 and 309.65 K.

**Table 1:** Force Field Parameters of Three-Site Non-Polarizable Water Models Used in This Work

	*r*_OH_	*k*_b_	θ	*k*_a_	σ_OO_	ϵ_OO_	*q*_H_
model	nm	kJmol^–1^nm^–2^	deg	kJmol^–1^rad^–2^	nm	kJmol^–1^	*e*
OPC3/LEW	0.097888	231,375	130.80	628	3.17327 × 10^–01^	5.54827 × 10^–01^	0.447585
OPC3	0.097888	345,000	109.47	383	3.17427 × 10^–01^	6.83691 × 10^–01^	0.447585
OPC3-B	0.095720	345,000	109.47	383	3.17427 × 10^–01^	7.74827 × 10^–01^	0.447585
SPCE	0.100000	345,000	109.47	383	3.16600 × 10^–01^	6.50155 × 10^–01^	0.423800
TIP3P	0.095720	502,416	104.52	628	3.15066 × 10^–01^	6.36270 × 10^–01^	0.417000

Once the chosen dielectric constant was reached, the LJ parameters were adjusted to reproduce the bulk water density and blood plasma at physiological conditions (309.65 K and 1 bar) as additional target properties. In this process, the original atomic charges and O–H distances were preserved. A cubic cell with the NPT ensemble and 500 water molecules was used to determine the dielectric constant and bulk density. MD trajectories were generated for 100 ns. For NVT simulations, an elongated box containing 2500 molecules, with dimensions *L*_*x*_ = *L*_*y*_ = 0.5*L*_*z*_, was used to reproduce surface tension, with MD trajectories of 20 ns long.

### Quantum Mechanics Calculations

In order to analyze the energy effects associated with opening the HOH angle, quantum-mechanical computations were performed. Using Density Functional Theory (DFT) and the *ModRedundant* tool implemented in Gaussian 16,^59^ an energy scan was carried out. Starting from the structure reported by Izadi et al. for their OPC3 model,^45^ the angle was varied in increments of 1° until the value obtained in the MD simulations was reached. In each step, energies and full orbitals were determined to provide detailed insight into its frontier orbitals. For this purpose, frontier molecular orbital theory was utilized to obtain the energy profiles.^60^ In this analysis, OH bond lengths were fixed. These calculations were conducted at the B3LYP/6–31 + G­(d) level of theory.^61–64^

### Validation and Assessment of the New Water Model

To validate the new model, three assessments were performed using MD simulations: two as a pure component and one as a solvating medium for eight helical peptides. The first involved evaluating five properties: dielectric constant, bulk density, blood surface tension, diffusion coefficient, and hydrogen bond (H-bond) formation at five temperatures (298.15, 304.15, 309.65, 314.15, and 323.15 K). Results were compared with experimental and OPC3 values. Second, its performance in reproducing nine physicochemical properties as a pure component was contrasted with four widely used three-point water models (OPC3, OPC3-B,^46^ SPC/E,^43^ and TIP3P^44^).

Finally, the third validation focused on evaluating its capacity to preserve peptide helical structures by measuring the percentage of helical conservation, the number of intramolecular H-bonds, and the folding/unfolding of the peptide structures. For this purpose, eight peptides with mainly helical structure were chosen: β-amyloid (PDB ID: 1IYT^65,66^ ), bacteriocin (1O83 ^67^), cathelicidin (2K6O^68^), granulysin (1L9L^69^), exenatide (7MLL^70,71^ ), liraglutide (4APD^72^), semaglutide (7KI0 ^73,74^ ), and tirzepatide (7FIM^75,76^ ). The peptide-water systems were prepared from their crystalline structures retrieved from the PBD server. Missing or incomplete residues were built using UCSF Chimera v.1.14 software,^77,78^ and their interaction parameters were obtained through the LigParGen server.^79,80^ Cubic simulation boxes were used to place the peptide at the center of the boxes, ensuring that the minimum distance between the boundaries and the peptide was greater than 2 nm. Then, the systems were solvated with the original and reparametrized water models, and the charge was neutralized by the addition of Cl^–^ and Na^+^ counterions at a concentration of 0.154 M.

### MD Simulations

All MD simulations were conducted using the GROMACS 2021 package and the OPLS-AA force field.^55–58^ The first step was to perform an energy minimization using the *steepest descent* algorithm for 50,000 steps, with only peptide structures in vacuum to achieve optimal results. A second energy minimization was performed considering the systems solvated by water and ions. Two equilibrium simulations were then performed in the NVT (50 ps) and NPT ensembles with position restraints in heavy atoms and a temperature of 323.15 K to ensure stability. The V-rescale thermostat^81^ was used for the NVT simulation, while the Nosé-Hoover thermostat^82^ and Parrinello–Rahman barostat^83^ were utilized for the NPT simulation. The compressibility factor was set at 4.5 × 10^–5^ 1/bar. The NPT equilibrium simulation trajectory was 3 ns, saving positions and velocities every nanosecond. These frames then served as initial structures in the production simulations of each replica.

The MD production trajectories were run for 100 (pure component), and 200 ns (peptide-water systems) in the isobaric–isothermal ensemble at 309.65 K. The trajectories were performed without position restraints. In all simulations, the equations of motion were integrated using a leapfrog integrator with a time step of 1 fs. Periodic boundary conditions (PBCs) were used in the *x*, *y*, and *z* directions. All MD simulations employed a particle mesh Ewald (PME) algorithm^84^ for long-range electrostatics with cubic interpolation. The cutoff for both the real part of the Ewald summation and the Lennard–Jones (LJ) interactions was set to 1.2 nm, with long-range corrections added for dispersion interactions. Additionally, a linear constraint solver (LINCS)^85^ with all bonds constrained was applied in all MD simulations.The Nosé–Hoover thermostat with time constant τ_T_ = 1.0 ps was used to couple the internal temperature. Isotropic pressure coupling was maintained at 1.0 bar using the Parrinello–Rahman barostat with a time constant τ_P_ = 2.0 ps. All production trajectories were saved every ten picoseconds. To increase statistical significance and validate conformational space sampling, two independent simulations were performed for each peptide-water system. A total of 400 ns was run for each system.

### Structure and Data Analysis

Several GROMACS modules were used to obtain statistical results. For water properties as a pure component, *gmx dipoles*, *gmx energy*, *gmx rdf*, and *gmx msd* modules were used to calculate the dielectric constant, density, radial distribution functions, and self-diffusion coefficient values, respectively. For peptide structures, the root-mean-square deviation (RMSD), root-mean-square fluctuation (RMSF), radii of gyration (RG), mean square displacement (MSD), solvent-accessible surface area (SASA) values were computed using *rms*, *rmsf*, *gyrate*, and *sasa* modules, respectively. The geometric criteria predetermined by GROMACS were used to calculate H-bonds, i.e., *r* ≤ *r*_HB_ = 0.35 nm and θ < θ_HB_ = 30°.^86^ The VMD software was used to calculate the percentage of H-bond occupancy along the production trajectories. The structural properties and stability indicators of the peptides were analyzed throughout the entirety of the molecular dynamics (MD) production trajectories. In contrast, the values presented in the tables are reported for the final 50 ns of the trajectories, during which the simulations stabilize. Structures were visualized using Visual Molecular Dynamics (VMD) and UCSF Chimera v.1.14,^77,78^ while the plots were generated with XMGrace.^87^ The energy corresponding to the configurational space of each peptide and the various water models was mapped based on RMSD and radius of gyration as variables. The *gmx sham* module generated the free energy landscape (FEL) data, which were visualized using Wolfram Mathematica 12.1.^88^

## Results and Discussion

The representation of the electrostatic environment in systems involving cell membranes has received little attention in computational simulation studies.^30^ However, the electrostatic forces play a fundamental role in the structural stability and interactions of biomolecules, and therefore in their physiological function.^89,90^ Water, the main component of this microsurrounding, provides the driving force that can modify the static and dynamic properties of these systems.^13,91^ Experimental and computational studies suggest that various properties of water, both structural and dynamic, are altered by the presence of lipids and organic cosolvents at the membrane interface, and that these alterations can propagate as far as approximately 2.4 nm from the cell membrane.^3,18,92,93^ In such conditions, the dielectric constant has been reported to range from 4 to 40.^13,23,24,94^ This decrease in surrounding electrostatics affects both the structure and interactions of macromolecules.^30^ Proper handling of electrostatic interactions is crucial in theoretical studies of systems that involve membrane environments. This study addresses the effect that a novel water model, specifically designed to represent low-polarity environments, produces on the properties and preservation of secondary structures of helical peptides.

### OPC3/LEW Model as a Pure Component

#### Building the New Water Model at 309.65 K

The structure and interaction parameters were obtained by performing MD simulations at physiological temperature (309.65 K). By varying the HOH angle, the dipole moment of the molecule was readjusted until a dielectric constant of 20 (130.8°) was reached (Figure 1a,b). This angle remained flexible across all simulations, with a restitution constant similar to that of the TIP3P model (*k*_θ_ = 628.02 kJ mol^–1^ rad^–2^),^44^ resulting in an average angle of 128.5°. The equilibrium distance for the OH bond remained constant and equal to that of the OPC3 model, with a restitution constant, *k*_B_, 33% lower (Table 1). With these parameters and using the dielectric constant as a stability criterion, the novel model exhibited rapid convergence and fluctuations of less than ±0.48, compared with the OPC3 model (±2.14). Although this new model aims to characterize only the electrostatic environment near the cell membrane, without considering the effect of cosolvents, the energy cost of transitioning to an open structure was calculated. For this purpose, electronic structure simulations were performed using DFT calculations at B3LYP/6–31 + G­(d) level of theory. Starting from the angle reported for the OPC3 model (109.47°) and that obtained by optimizing the structure level (105.49°), an energy sweep was performed until the angle proposed for the OPC3/LEW model was reached. The analyses show considerable energy barriers of 37.29 (OPC3) and 36.32 kJ/mol (optimized) (Figure S1). Given that the average thermal energy of a water molecule at physiological temperature is approximately 7.72 kJ/mol (based on the six degrees of freedom of an isolated water molecule), these energy values highlight a weakness of the OPC3/LEW model: the opening of the HOH angle would not occur spontaneously.

In this regard, calculations of the frontier molecular orbitals (FMO) show that increasing the angle raises the HOMO (Highest Occupied Molecular Orbital) and LUMO (Lowest Unoccupied Molecular Orbital) energies, thereby decreasing the energy gap by approximately 8.5 kJ/mol relative to the optimized structure (Table 2). Furthermore, when comparing the FMO energies of the optimized water structure with those of the open structure, the greatest effect is observed in the HOMO–1 and HOMO–2 orbitals. For HOMO–1, the energy of the OPC3/LEW model increases by approximately 0.0423 au (atomic units), while it decreases in HOMO–2 by approximately 0.0347 au (Figure 1c and Table S1). The destabilization of the orbitals and the redistribution of charge reduce the electrostatic dipole by 21.8%, weakening the electrostatic attractions without altering the chemical identity of the system. The result would be a more reactive molecule, which is reflected in its lower hardness (η). However, the OPC3/LEW structure also has the lowest electronegativity (χ) and electrophilicity (ω) values, which would decrease its ability to interact through electrostatic interactions. Its effect would be to prevent the formation of rigid hydration layers, favoring a more fluid and nondisruptive interfacial environment, properties desired for this model.

**Table 2:** DFT Results^a^,^b^

Water	Angle	DM	HOMO	LUMO	*E*_gap_	η	*S*	χ	ω
model	(Degrees)	(Debye)	(a.u.)	(a.u.)	(a.u.)	(a.u.)	(a.u.)	(a.u.)	(a.u.)
Opt.	105.49	2.25	–0.31926	0.02489	0.34415	0.17208	5.81142	0.14719	0.06295
OPC3/LEW	131.80	1.76	–0.31397	0.02695	0.34092	0.17046	5.86648	0.14351	0.06041
OPC3	109.47	2.18	–0.31758	0.02413	0.34171	0.17086	5.85292	0.14673	0.06300

a Structure and reactivity parameters of water molecules.

b DM: Dipolar moment; HOMO: Highest occupied molecular orbital; LUMO: Lowest unoccupied molecular orbital; η: Chemical hardness; *S*: Global softness; χ: Electronegativity; ω: Electrophilicity index.

The electrostatic decay of the OPC3/LEW model can be observed by analyzing the electrostatic potential surfaces (ESPS, Figure 1c in the inset). While the optimized model shows the known dipolar character of water, the OPC3/LEW model exhibits a distortion due to the separation of the hydrogen atoms. The consequence is a decrease in nucleophilic character around the oxygen atom (red in the ESPS) despite the 5.28% negative increase in its atomic charge (Figure S1). On the other hand, an increase in electrophilic character is observed around the hydrogen atoms due to structural rearrangement (blue color), resulting in a neutral region between them. This distortion in its geometry would impair its ability to form intermolecular H-bonds with other chemical entities or to modify the local structure around each molecule, thereby affecting its H-bond network.^95^

Because the local intermolecular structure of bulk water depends on its density,^96^ new MD simulations were performed to adjust the Lennard-Jones interaction parameters (σ_LJ_ and ϵ_LJ_) to reproduce the density at 309.65 K (993.7 kg/m^3^). Once this was achieved, the last 10 ns of the MD trajectories were used to evaluate the tetrahedral character of the new model and to compare it with that obtained with the OPC3 model (Figure 1d) under the same conditions. Based on the work of Chau et al.,^97^ the angle order (*S*_g_) and distance order (*S*_k_) parameters were calculated using the *gmx hydorder* tool with oxygen as the reference atom. According to these authors, the perfect tetrahedral structural arrangement is achieved as these parameters tend to zero. In their study, they reported values for *S*_g_ = 0.100 and *S*_k_ = 0.0013 using the SPC/E water model at 320 K. Taking these values as a reference, the results show that at physiological temperature, both OPC3 models exhibit local tetrahedral structures, with the LEW model showing errors of only 2% and 0% for *S*_g_ and *S*_k_, respectively. In addition, considering the oxygen atoms as geometrical points, distance and angular distributions exhibit average values of 105.5° for the O–O–O angle, and 0.28 nm for the O–O length (Figure 1d).

In this sense, the local structure of water can also be inferred from its radial distribution function, g­(*r*). From the oxygen–oxygen g­(*r*_O–O_) calculation, a coordination number (*C_n_*) of 4.36 was obtained for the new model, very close to the experimental value reported by Skinner et al. (4.3 ± 2) at 307 K using high-energy X-ray diffraction techniques.^98^ In the OPC3 model, the value of *C*_*n*_ was 4.54 (Figure S2, and Table S2). Figure 1e shows the g­(*r*_O–O_) values obtained for the OPC3/LEW and OPC3 models compared with the experimental value. Both OPC3 models overestimate the maximum of the first experimental peak by almost 20%. However, due to its open structure and changes in LJ parameters, the new model allows greater packing around the central molecule, placing this peak at 2.72 Å from the central oxygen. In the case of OPC3, the peak is at 2.76 Å, while the experimental position is 2.80 Å. This better fit in the g­(*r*_O–O_) of the OPC3 model is also reflected in the height and position of the second and third peaks, in agreement with the results reported by Izadhi et al. at 298 K in the design of this model.^45^ Notably, although the maximum of the second peak is shifted to the right by 0.19 Å relative to the experimental value, their minima agree, better reproducing the number of molecules within the second solvation layer (19) than OPC3 (20). Experimental and simulation studies suggest that the number of molecules ranges between 12 and 16.^99,100^ Furthermore, Rognoni et al. showed, using semiclassical spectroscopy calculations, that 16 molecules in the second solvation layer constitute the minimum aggregate required to solvate each central water molecule.^100^

#### Effect of the Temperature on Structural and Thermodynamic Properties of the OPC3/LEW Model

Although the main goal of the OPC3/LEW model is to reproduce the low electrostatic environments at physiological temperatures, its behavior in a range of temperatures was analyzed. To do this, additional MD simulations were performed at 298.15, 304.15, 314.15, and 323.15 K, at a constant pressure of one bar. In addition to its local structure, five properties were assessed: dielectric constant, density, surface tension, diffusion coefficient, and its ability to form intermolecular H-bonds. Results were compared with experimental data and those obtained with the OPC3 model (Figure 2).

**Figure 2:**
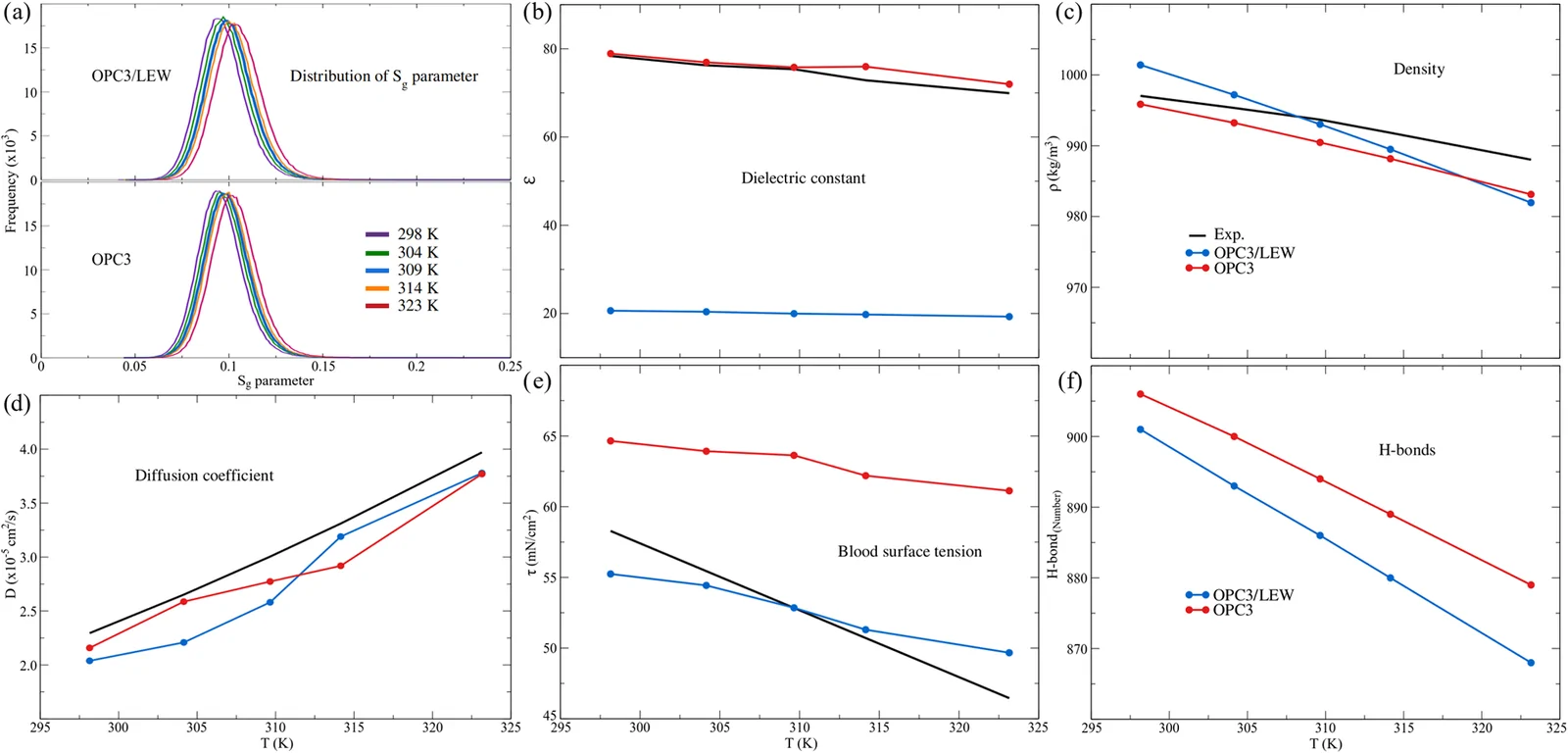
Target properties used for assessing the performance of the water model as a pure component as a function of temperature. (a) Tetrahedral angle parameter, (b) Dielectric constant, (c) bulk density, (d) surface tension, (e) diffusion coefficient, and (f) H-bond formation.

About the local structure, the distributions of *S*_g_ and *S*_k_ parameters show a temperature dependence (Figures 2a, and S2a); that is, as the temperature increases, the tetrahedral arrangement becomes distorted. Thus, at 323.15 K, the values of *S*_g_ and *S*_k_ were 0.104, 0.0014 (OPC3/LEW) and 0.102, 0.0011 (OPC3), which are close to those obtained by Chau et al. for the SPC/E model (0.100, 0.0013) at 320 K.^97^ The calculated values can be found in Table S2. The same trend has been observed and discussed in previous studies using other water models, employing QM and MM simulations.^95,97,101,102^ In this regard, the study by Chatterjee et al. is particularly interesting.^103^ They analyzed various properties of the SPC/E model, including tetrahedrality and the radial distribution function (*rdf*), as the HOH angle was varied from 60° to 120° at different temperatures. Based on the translational and tetrahedral order parameters (τ and *q* ^104–106^ ), the authors showed that at angles greater than 100°, water can recover a tetrahedral local environment. Moreover, at an angle of 120°, the tetrahedral configuration is preserved, although the order parameters diminish as the temperature increases. On the other hand, the coordination number shows a slight rise in both OPC3 models (Table S2), with the LEW model showing a lower increase (4.2%) than the OPC3 model (8.6%) at 298–323 K. However, Skinner et al. experimentally showed that *C*_*n*_ is constant over that temperature range, with a value of 4.3 ± 2,^98^ so the LEW model best fits those data.

Concerning the dielectric constant, Figure 2b shows that the OPC3 model accurately reproduces the experimental values over the analyzed temperature range, whereas the LEW model does not. Although the behavior was as expected when setting a target value of 20, the interest in evaluating this property lay in determining whether this model maintained well-behavior during MD simulations as the temperature varies. Thus, the results show that increasing the angle does not modify the negative slope of the experimental results. Although the decrease is smaller (*m* = −0.05, compared to *m* = −0.34 for the experimental values), the same trend is observed in solvents such as acetone, whose dielectric constant is 20.49 and 17.96 at 298 and 323 K, respectively (*m* = −0.10).^107^

For bulk water density, the results show that at temperatures close to physiological, the new model agrees with the experimental curve (Figure 2c). However, reproducibility is lost as temperature moves away from this point. At low temperatures, its open structure and the decrease in the σ_LJ_ parameter lead to greater molecular packing, observed in the g­(*r*_O–O_) model (Figure S2), reaching a value greater than 1001 kg/m^3^ at 298 K. Similar trends were obtained by Chatterjee et al., with open-angle variants of the SPC/E model (115° and 120°).^103^ On the other hand, the density decay at high temperatures is mainly due to a reduced attractive interaction caused by the change in ϵ_LJ_, it being even lower than that obtained with OPC3. When analyzing density as a function of pressure, similar behavior is observed at physiological temperature (Figure S3a) as well as at 298.15 and 323.15 K (Figure S3b). Nonetheless, computational studies of biological systems indicate that water at the cell membrane interface does not maintain bulk density. Calero et al., using molecular dynamics with the TIP3P model and the DMPC membrane at 303 K, showed that the density increased in the first hydration layer (∼0.5 nm).^108^ Moreover, Martelli et al., using a modified TIP3P model, concluded that the water density recovered its bulk value at distances greater than one nanometer (>1 nm).^93^

Furthermore, changes in ϵ_LJ_ modify important properties in the LEW model. By decreasing the O–O interaction energy, the diffusion of molecules is lower at high densities due to optimized packing (greater tetrahedricity, Figure 2d).^109^ Once the target temperature is reached, the density decreases, thereby increasing molecular diffusion in the model. This behavior is also observed in surface tension (Figure 2e). However, optimizing these parameters enables the OPC3/LEW model to accurately reproduce the surface tension of the bloodstream, as shown in the experimental curve. This latter property was considered a primary objective in the development of OPC3/LEW, given its importance in interfacial phenomena at the cell membrane.^110^ The interfacial tension can affect the structural properties of lipid membranes,^111,112^ drug interactions,^113^ and protein and peptide denaturation,^52,114^ leading to a poor representation of traditional water models.^52,115^

A final property of interest for the construction of the new model was the formation of intermolecular H-bonds. Experimental and computational studies have shown that in a low electrostatic environment, intermolecular interactions between proteins and water molecules decrease, promoting the formation of intramolecular bonds within proteins.^24,30^ Therefore, one of the goals of the new model was to develop a less aggressive approach toward biomolecules. As a pure component, a decrease in these interactions was expected due to the force-field reparametrization. The results are shown in Figure 2f. As expected, the graph shows a lower formation of this type of interaction as the temperature increases, compared to the values obtained with the OPC3 model. Although the decrease is small (1%, corresponding to about 8 bonds), the simulation cells contained 500 molecules. In systems involving membranes and proteins, the number of water molecules can reach tens of thousands, providing a more accurate representation of the environment near the cell membrane. Nonetheless, the outcomes show that at physiological temperature, OPC3/LEW is capable of reproducing the target water property values, even outperforming the OPC3 model. However, as the temperature deviates from this point, its performance declines.

#### Benchmark of Bulk Water Properties against Other Popular Rigid Three-Site Water Models

How well does the OPC3/LEW model compare with other three-site water models for calculating physicochemical properties? To validate the performance of the new model, MD simulations were performed at 298.15 and 309.65 K for 100 ns for the OPC3-B, SPC/E, and TIP3P models, in addition to OPC3. The evaluated properties are shown in Table 3 and their normalized relative errors in Figure 3.

**Figure 3:**
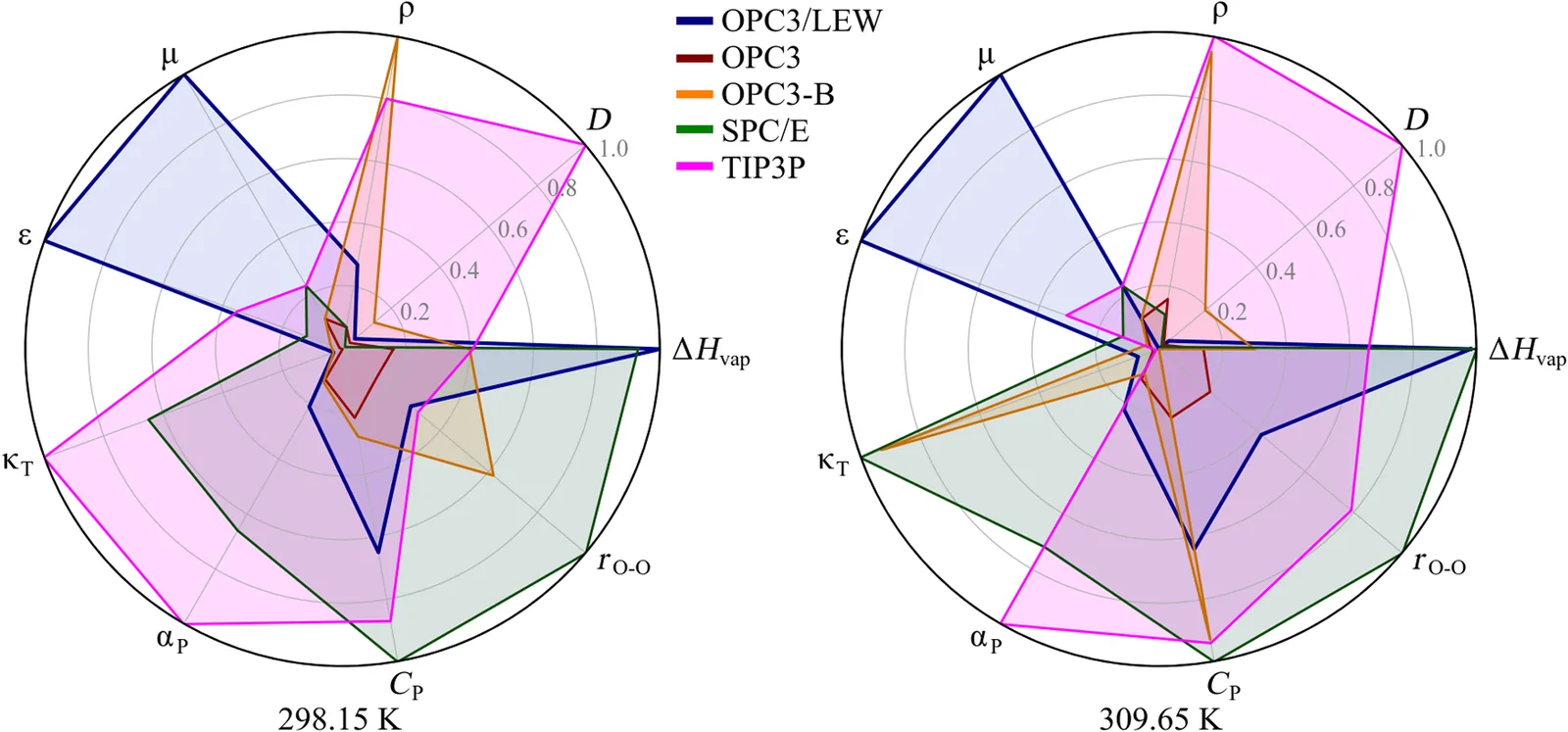
Normalized relative errors of the calculated physicochemical properties. The errors were calculated using experimental values at the analyzed temperatures as references. Normalization was performed using the maximum error value for each property. ϵ = dielectric constant; μ = dipole moment; ρ = density; *D* = self-diffusion coefficient; Δ*H*_vap_ = enthalpy of vaporization; *r*_O–O_ = radial distribution function for oxygen pairs; *C*_P_ = heat capacity at constant pressure; α_P_ = coefficient of thermal expansion; κ_T_ = isothermal compressibility.

**Table 3:** Properties Calculated Using Rigid Three-Site Models^a^

Water		μ	ρ	*D*	Δ*H*_vap_	*r*_O–O_	*C*_P_	α_P_	*K*_T_	ARE
Model	ϵ	(D)	(g/cm^3^)	(m^2^/s)	(kJ/mol)	(Å)	(J/(mol·K))	(10^–4^/K)	(10^–6^/bar)	%
298.15 K										
Exp.	78.40	2.5–3.0	0.997	2.30	44.02	2.80	75.31	2.56	45.30	-
OPC3/LEW	20.63	1.84	1.001	2.04	38.41	2.72	129.62	9.19	49.09	51.87
OPC3	78.89	2.43	0.996	2.16	44.89	2.76	94.14	6.07	45.95	19.62
OPC3-B	80.60	2.43	0.982	3.03	46.23	2.98	98.99	6.17	49.57	25.90
SPC/E	71.38	2.35	0.998	2.36	49.25	3.09	159.03	23.08	137.54	128.47
TIP3P	98.42	2.35	0.985	7.83	46.32	2.71	148.45	33.84	186.38	212.45
309.65 K										
Exp.	74.36	2.50	0.993	3.04	43.56	2.80	73.30	3.39	44.57	-
OPC3/LEW	19.97	1.84	0.993	2.58	37.49	2.72	127.19	10.04	52.15	46.46
OPC3	75.79	2.43	0.990	2.77	44.39	2.76	92.51	6.65	46.59	16.02
OPC3-B	76.55	2.43	0.975	5.21	45.40	2.80	151.63	6.12	140.80	54.05
SPC/E	67.65	2.35	0.991	2.89	49.75	2.99	157.95	25.43	148.25	115.49
TIP3P	91.34	2.35	0.974	14.74	47.66	2.65	152.46	33.84	45.30	159.81

a MD simulations were performed for 100 ns at 298.15 and 309.65 K, both at 1 bar.

With average relative errors (AREs) of 19.6% at 298.15 K and 16.02% at 309.65 K, the data indicate that the OPC3 model outperforms the other models in reproducing the evaluated properties at both temperatures (Table 3). For this model, the properties with the lowest accuracy were the coefficient of thermal expansion (α_P_) and *C*_P_, with maximum errors of 137.1% and 26.2%, respectively. OPC3 was developed by Izadi and Onufriev,^45^ with parameter optimization based on the important role of charge distribution in describing the electrostatic properties of the water molecule, which are key to mimicking its behavior in the liquid phase. Derived from this model, the OPC3-B and OPC3/LEW models were the second-best performers, at 298.15 and 309.65 K, respectively. For the OPC3-B model, the normalized relative error graphs show that density was the least accurate property at both temperatures (Figure 3).

Regarding the OPC3/LEW model, the data indicate that although changes in structure and parameters were expected to affect property calculations, it is not the worst-performing model (Table 3). Based on the ARE values, OPC3/LEW outperforms the traditional SPC/E and TIP4P models at the temperatures analyzed, surpassed only by OPC3-B at 298.15 K. Since it was parametrized for physiological temperature (309.65 K), OPC3/LEW better describes properties such as density, diffusion, *r*_O–O_, and *k*_T_. However, at both temperatures, *C*_P_ and α_P_ showed the largest relative errors, making the model unsuitable for systems where accurate descriptions of these properties are crucial to the desired results. The Δ*H*_vap_ results are noteworthy: at both temperatures, its values were the lowest among all models. This accurately describes the behavior of a low-polarity solvent, in which the energy required to transition to the gas phase depends largely on intermolecular interactions. In OPC3/LEW, hydrogen-bonding interactions decrease, leading to an underestimation of this property.

As the calculated results show, the models with the worst performance were SPC/E and TIP3P. Despite their known shortcomings in reproducing thermodynamic and structural anomalies of pure water, the TIP3P and SPC/E models remain relevant due to their low computational cost and the fact that several force fields have been developed using them.^12,116^

### OPC3/LEW Model as a Solvating Medium in Helical Peptide Systems

As mentioned above, the LEW model was developed to preserve or promote helical structures in low-electrostatic environments, such found near a cell membrane surface.^14,24^ Therefore, as a final validation, the model was tested as a solvating medium for eight peptides with primarily helical structures (Figure 4a). Using MD simulations, the performance of the LEW model was evaluated by calculating its stability indicators and its ability to preserve secondary structures and promote intramolecular H-bonds. Two MD simulations with 200 ns trajectories were run for each peptide at physiological temperature; therefore, the values presented are the averages of both simulations. The results were compared with those obtained in the previously analyzed water models. To facilitate reading and understanding, the results for each water model are presented with a color code: OPC3/LEW, blue; OPC3, red; OPC3-B, orange; SPC/E, green; and TIP3P, magenta.

**Figure 4:**
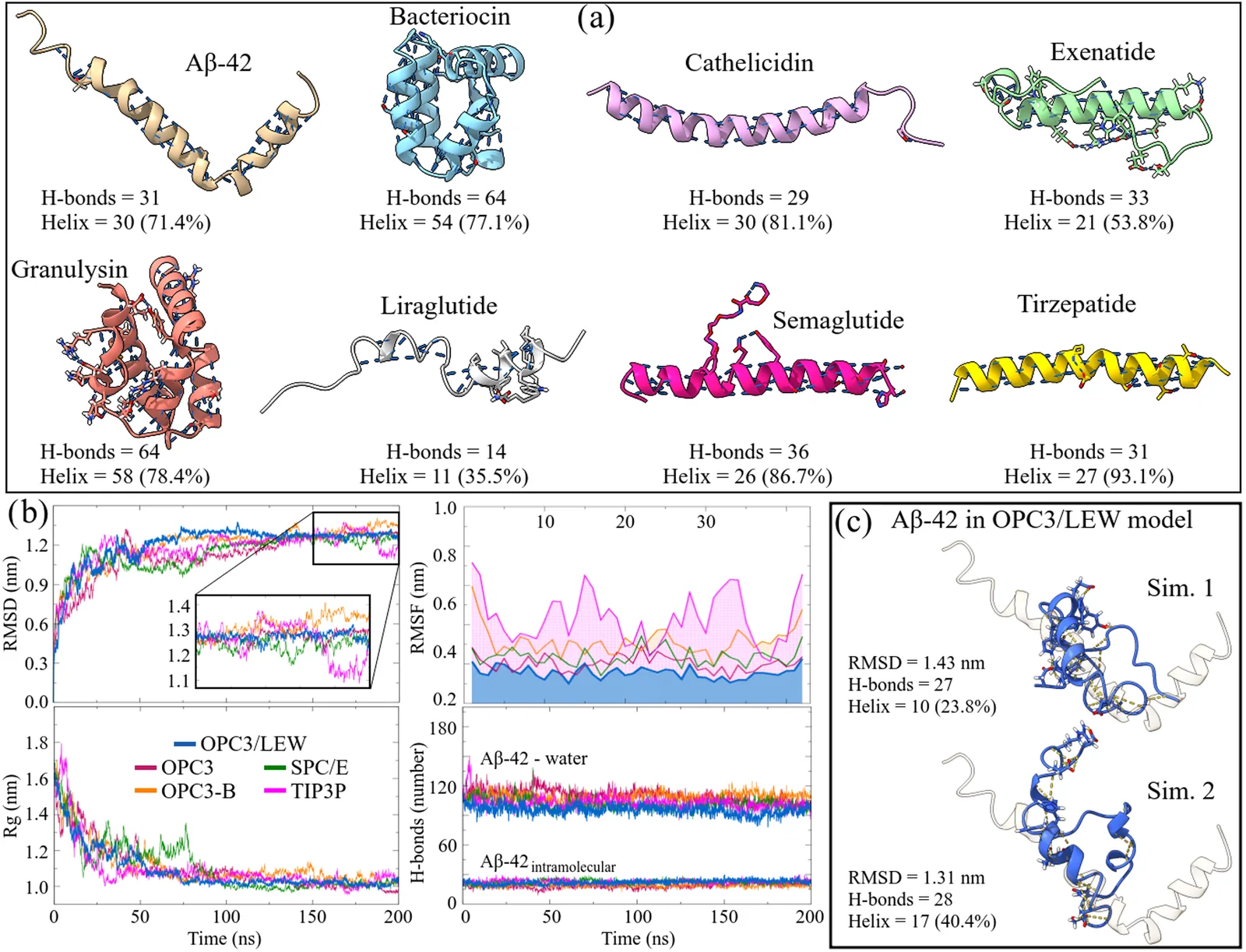
Peptides used in the validation of the OPC3/LEW water model: (a) β-amyloid (42 residues), bacteriocin (70 residues), cathelicidin (37 residues), exenatide (38 residues), granulysin (74 residues), liraglutide (31 residues), semaglutide (30 residues), and tirzepatide (29 residues). The number of intramolecular H-bonds and helical structure percentages are indicated for each peptide. (b) Stability indicators of the Aβ-42 structure using the five water models studied. Each curve represents the average of the two simulations performed. (c) Structural alignment and analysis of the final structures (original and replicate) with respect to the experimental structure, obtained from the MD trajectories for the Aβ-42 peptide.

#### MD Simulations Exhibit a Rapid Convergence and More Stable Structures Using the OPC3/LEW Model as a Solvent

The relevance of secondary structures in integral membrane proteins has not yet been fully elucidated, largely because they are difficult to analyze in their lipid environments.^3,5,117^ Nonetheless, several diseases are associated with conformational changes or protein misfolding, leading to proteins being far from their native structure.^14^ While it is well-known that the structure and folding of water-soluble proteins are dominated by the hydrophobic effect,^15,118^ this is not the case for proteins in the cell membrane, where electrostatic decay in the environment is key to modulate their interactions. Various experimental and simulation studies have shown that under such conditions, secondary structures, particularly helical ones, are favored.^14,24^ Experimentally, the use of mixtures of water with low-polarity solvents, such as alcohols,^119,120^ to mimic low-dielectric environments and analyze their effects on stabilizing secondary structures in peptides and proteins is well established.^14,24,119,120^ However, performing such studies by computational means would be expensive, and additionally, several widely used models incorrectly describe the dielectric constant of bulk water.

Figure 4a shows the eight peptides used to evaluate the new water model. Their structures are composed primarily of hydrophobic residues, an important characteristic of helical structures in membrane proteins,^3,121^ ranging from 39.2% (granulysin) to 67.1% (bacteriocin). Furthermore, they exhibit amphipathic properties,^122^ making their study relevant, as they may interact with cell membranes (Figure S4, and Table S3).^123,124^ An example is cathelicidin, whose amphipathic properties result from its biological function in the immune system, enabling it to penetrate the membranes of pathogens.^68^ Except for liraglutide, the analyzed peptides have a secondary structure composed of more than 70% α helices. Furthermore, both bacteriocin and granulicin form tertiary structures, which allowed for the evaluation of the effect of the new model on the destabilization of their conformations. To assess convergence in the simulations, two stability descriptors were used: RMSD and RMSF. The average values for the last 50 ns of the MD trajectories are shown in Table S4. The analysis of these descriptors reveals one of the main advantages of using the OPC3/LEW model as a solvating medium: the speed at which peptides attain structural stability, thereby accelerating simulation convergence. RMSD plots indicate that, with the OPC3/LEW model, the equilibrium plateau is reached around 50 ns in the MD simulations. Furthermore, it is the solvent with the greatest convergence in the last 50 ns of the trajectory, with standard deviation values no greater than ±0.03 nm (insets in RMSD plots, Figures 4b and S5a–g). The greatest convergence was observed with bacteriocin and granulysin (std. dev. = ±0.01 nm), where the additional stability is due to their tertiary structures, allowing them to penetrate cell membranes.^125–127^ In contrast, cathelicidin, with the highest amphipathicity value, was the most unstable peptide, exhibiting the greatest fluctuation in its average RMSD values (Table S4). Nonetheless, this behavior is expected, as cathelicidin has been experimentally shown to be flexible and disordered in aqueous solutions.^68,128^ This explains the lack of convergence when using the other models analyzed.

The structural stability promoted by the OPC3/LEW model is further evidenced by comparing the per-residue fluctuations in the analyzed systems. The RMSF plots and average values indicate that, with this model, the deviations of the average atomic positions do not exceed 0.25 nm, which are smaller than those calculated with the other water models. These results reveal that the convergence in the simulations is a consequence of reduced solvent-peptide interactions, leading to fewer H-bonds between them and promoting intramolecular interactions. In that sense, the new model would reproduce one of the proposals that identify this type of interaction as the driving force behind folding in membrane proteins.^15^ The analyses also showed that SPC/E is the model that most closely approximates OPC3/LEW results. Both RMSD and RMSF calculations show that the systems tend to stabilize over time and exhibit per-residue fluctuations only slightly higher than those obtained with the OPC3/LEW model. Importantly, the SPC/E dielectric constant was the lowest among the models compared (ϵ = 67.65 at 309.65 K, Table 3). These findings reinforce the idea that the electrostatic environment can affect peptide properties and is an important factor for accurate representation in computer simulations.

#### OPC3/LEW Model Exhibits the Greatest Number of Intramolecular H-Bonds in MD Simulations

In a previous study, Aguilar-Pineda et al. used two low electrostatic water models to demonstrate their effects on the structures of membrane proteins and peptides.^30^ By modifying the interaction parameters of two flexible models,^53,54^ they observed that the outcome models promoted the formation of intramolecular H-bonds, leading to more compact protein structures. While their results reproduced the folding-driving force in nonpolar solvents, the preservation of helical structures in the evaluated peptides did not represent a significant improvement over that obtained with the original models.

Given the importance of these structural descriptors, several analyses were conducted to assess the performance of the OPC3/LEW model in preserving peptide structures. Peptide folding was analyzed by measuring the total radius of gyration, a measure of structural compaction. Furthermore, since H-bonds mediate folding, their numbers were calculated intramolecularly and intermolecularly (peptide-water and water–water). *R*_g_ and H-bonds plots along MD trajectories are shown in Figures 4b and S5. Finally, the preservation of helical structures was assessed by analyzing the number of residues forming structural motifs using the Generate tool of the PDBSum server.^129^ These calculations were performed on the final structures (200 ns) of each replicate across all water models, and their average values are reported in Table 4.

**Table 4:** FEL Analysis^d^,^e^

Water	^a^Radius of	^b^H-bonds				Final structures			^e^FEL analysis			Min.Energy structures	
Model	gyration	Intra	Pep-wm	wm-wm		^b^H-bonds	^c^Total helix		Area	Energy		^b^H-bonds	^c^Total helix
Aβ-42	1.63	31	-	-		31	30 (71.4)					31	30 (71.4)
OPC3/LEW	1.02 ± 0.01	24 ± 2	91 ± 5	21943 ± 38		27.5 ± 0.5	14 (33.3)		0.92 ± 0.05	–16.65 ± 2.62		30.0 ± 0.0	9.0 (21.4)
OPC3	1.00 ± 0.02	20 ± 2	104 ± 4	22096 ± 34		27.0 ± 6.0	5 (11.9)		0.93 ± 0.12	–13.95 ± 2.05		22.8 ± 1.7	7.3 (17.3)
OPC3-B	1.04 ± 0.02	19 ± 2	111 ± 4	21574 ± 37		23.0 ± 3.0	6 (14.3)		1.04 ± 0.06	–14.20 ± 0.28		24.7 ± 1.8	6.3 (15.1)
SPC/E	1.01 ± 0.01	22 ± 2	101 ± 4	21952 ± 35		29.5 ± 2.5	5 (11.9)		1.19 ± 0.11	–15.30 ± 1.27		26.5 ± 0.5	7.0 (16.7)
TIP3P	1.02 ± 0.03	23 ± 2	99 ± 4	20339 ± 44		31.5 ± 0.5	4 (9.5)		1.23 ± 0.12	–14.20 ± 0.99		28.3 ± 1.4	4.0 (9.5)
Bacteriocin	1.09	64	-	-		64	54 (77.1)					64	54 (77.1)
OPC3/LEW	1.09 ± 0.00	40 ± 3	113 ± 3	21608 ± 37		54.5 ± 2.5	46 (65.7)		0.02 ± 0.01	–10.02 ± 0.25		50.5 ± 2.0	40.8 (58.2)
OPC3	1.10 ± 0.01	39 ± 2	121 ± 4	21752 ± 36		45.5 ± 4.5	33 (47.1)		0.02 ± 0.01	–9.97 ± 0.18		49.0 ± 3.0	34.5 (49.3)
OPC3-B	1.11 ± 0.01	38 ± 2	124 ± 4	21239 ± 38		46.0 ± 3.0	33 (47.1)		0.02 ± 0.01	–10.20 ± 0.28		45.3 ± 2.2	33.7 (48.1)
SPC/E	1.10 ± 0.01	38 ± 2	122 ± 4	21610 ± 37		47.0 ± 2.0	32 (45.7)		0.02 ± 0.00	–10.35 ± 0.07		53.3 ± 1.8	40.3 (57.6)
TIP3P	1.11 ± 0.01	38 ± 3	120 ± 4	20024 ± 43		47.5 ± 4.5	27 (38.6)		0.02 ± 0.01	–10.10 ± 0.14		44.7 ± 2.2	34.0 (48.6)
Cathalecidin	1.66	29	-	-		29	30 (81.1)					29	30 (81.1)
OPC3/LEW	1.15 ± 0.01	25 ± 2	94 ± 4	41377 ± 52		31.0 ± 5.0	18 (48.6)		0.81 ± 0.28	–14.55 ± 0.35		31.0 ± 2.3	17.0 (45.9)
OPC3	1.30 ± 0.06	20 ± 2	114 ± 4	41658 ± 49		27.0 ± 1.0	15 (40.5)		0.88 ± 0.03	–10.90 ± 0.99		30.0 ± 1.0	15.0 (40.5)
OPC3-B	1.17 ± 0.03	21 ± 2	112 ± 4	40677 ± 52		26.0 ± 5.0	11 (29.7)		1.12 ± 0.16	–12.45 ± 3.18		30.0 ± 5.5	12.3 (33.3)
SPC/E	1.07 ± 0.03	20 ± 2	110 ± 5	41386 ± 49		27.5 ± 0.5	13 (35.1)		1.12 ± 0.16	–13.65 ± 1.20		23.7 ± 0.3	11.7 (31.5)
TIP3P	1.04 ± 0.03	23 ± 2	105 ± 5	38350 ± 60		27.5 ± 2.5	14 (37.8)		1.17 ± 0.17	–13.50 ± 0.71		32.0 ± 1.5	13.0 (35.1)
Exenatide	1.16	33	-	-		33	21 (53.8)					33	21 (53.8)
OPC3/LEW	1.11 ± 0.02	27 ± 2	91 ± 5	12292 ± 28		35.5 ± 0.5	20 (51.3)		0.23 ± 0.09	–12.45 ± 0.07		37.7 ± 1.8	17.7 (46.5)
OPC3	0.94 ± 0.01	23 ± 2	98 ± 4	12379 ± 28		30.0 ± 1.0	13 (33.3)		0.45 ± 0.10	–14.50 ± 0.14		29.5 ± 2.5	14.0 (36.8)
OPC3-B	1.16 ± 0.06	22 ± 2	104 ± 7	12083 ± 29		29.5 ± 3.5	12 (30.8)		0.43 ± 0.09	–11.40 ± 0.14		34.0 ± 2.0	14.5 (38.2)
SPC/E	1.07 ± 0.02	21 ± 2	101 ± 5	12295 ± 27		29.5 ± 2.5	14 (35.9)		0.26 ± 0.03	–12.15 ± 0.35		30.8 ± 2.4	16.0 (42.1)
TIP3P	1.09 ± 0.02	25 ± 2	90 ± 4	11393 ± 32		31.5 ± 1.5	12 (30.8)		0.30 ± 0.13	–11.00 ± 0.03		36.5 ± 0.5	16.0 (42.1)
Granulysin	1.20	64	-	-		64	58 (78.4)					64	58 (78.4)
OPC3/LEW	1.19 ± 0.01	52 ± 3	168 ± 7	22883 ± 39		63.0 ± 3.0	51 (68.9)		0.02 ± 0.01	–10.85 ± 0.21		71.0 ± 2.0	45.5 (61.5)
OPC3	1.20 ± 0.01	54 ± 3	184 ± 5	23027 ± 38		75.0 ± 2.0	42 (56.8)		0.02 ± 0.01	–10.60 ± 0.28		66.5 ± 2.5	43.2 (58.3)
OPC3-B	1.20 ± 0.01	52 ± 3	188 ± 5	22482 ± 40		63.5 ± 1.5	41 (55.4)		0.03 ± 0.00	–10.90 ± 0.42		66.7 ± 2.1	39.7 (53.6)
SPC/E	1.20 ± 0.01	55 ± 3	185 ± 5	22877 ± 36		70.0 ± 4.0	39 (52.7)		0.02 ± 0.01	–11.15 ± 0.21		68.0 ± 1.3	43.6 (59.0)
TIP3P	1.19 ± 0.02	52 ± 3	184 ± 6	21193 ± 46		69.5 ± 0.5	40 (54.1)		0.02 ± 0.01	–10.21 ± 0.41		64.7 ± 2.2	39.0 (52.7)
Liraglutide	1.38	14	-	-		14	11 (35.5)					14	11 (35.5)
OPC3/LEW	0.97 ± 0.01	19 ± 2	83 ± 4	31700 ± 46		22.5 ± 1.5	9 (29.0)		0.57 ± 0.01	–15.35 ± 0.35		21.8 ± 1.8	7.5 (25.0)
OPC3	0.95 ± 0.02	17 ± 2	90 ± 4	31922 ± 43		18.0 ± 4.0	7 (22.6)		0.99 ± 0.09	–13.40 ± 0.57		20.0 ± 2.1	8.3 (27.8)
OPC3-B	1.00 ± 0.04	19 ± 2	85 ± 4	31168 ± 46		24.0 ± 2.0	8 (25.8)		1.01 ± 0.13	–14.20 ± 1.27		21.5 ± 2.5	3.5 (11.7)
SPC/E	0.95 ± 0.02	20 ± 2	84 ± 5	31715 ± 66		22.0 ± 3.0	8 (25.8)		0.59 ± 0.00	–14.95 ± 1.20		21.0 ± 2.0	6.5 (21.7)
TIP3P	0.93 ± 0.03	23 ± 2	76 ± 4	29385 ± 52		26.5 ± 0.5	7 (22.6)		0.72 ± 0.01	–14.20 ± 2.97		23.0 ± 3.0	4.0 (13.3)
Semaglutide	1.52	36	-	-		36	26 (86.7)					36	26 (86.7)
OPC3/LEW	0.99 ± 0.01	24 ± 2	79 ± 3	29732 ± 42		30.5 ± 1.5	12 (40.0)		0.72 ± 0.08	–14.90 ± 0.57		25.5 ± 2.5	8.0 (27.6)
OPC3	1.03 ± 0.05	18 ± 2	91 ± 4	29931 ± 40		18.5 ± 1.5	5 (16.7)		0.78 ± 0.01	–15.20 ± 1.56		20.0 ± 0.0	5.0 (17.2)
OPC3-B	1.17 ± 0.05	15 ± 2	99 ± 5	29221 ± 45		15.5 ± 1.5	5 (16.7)		0.82 ± 0.10	–12.45 ± 0.92		23.0 ± 2.4	7.0 (24.1)
SPC/E	1.00 ± 0.01	21 ± 2	82 ± 3	29740 ± 43		20.0 ± 3.0	5 (16.7)		0.81 ± 0.23	–14.20 ± 0.14		22.0 ± 0.0	7.0 (24.1)
TIP3P	1.02 ± 0.03	18 ± 2	90 ± 4	27545 ± 50		24.5 ± 0.5	7 (23.3)		0.76 ± 0.04	–12.75 ± 1.20		21.5 ± 2.0	7.5 (25.9)
Tirzepatide	1.34	31	-	-		31	27 (93.1)					31	27 (93.1)
OPC3/LEW	0.95 ± 0.01	19 ± 2	65 ± 3	24334 ± 42		22.5 ± 0.5	8 (27.6)		0.59 ± 0.09	–12.95 ± 0.35		25.5 ± 0.5	9.0 (31.0)
OPC3	0.97 ± 0.04	14 ± 1	76 ± 3	24500 ± 38		18.0 ± 3.0	8 (27.6)		0.53 ± 0.04	–12.55 ± 2.33		15.7 ± 1.0	10.3 (35.6)
OPC3-B	0.99 ± 0.07	13 ± 2	75 ± 3	23922 ± 38		17.5 ± 0.5	4 (13.8)		0.93 ± 0.04	–12.85 ± 0.49		18.0 ± 1.1	8.4 (29.0)
SPC/E	0.97 ± 0.02	14 ± 2	74 ± 4	24338 ± 39		17.5 ± 0.5	9 (31.0)		0.55 ± 0.17	–14.35 ± 0.78		17.3 ± 1.3	10.0 (34.5)
TIP3P	0.85 ± 0.01	15 ± 2	70 ± 3	22545 ± 45		18.5 ± 0.5	11 (37.9)		0.77 ± 0.08	–15.80 ± 1.41		20.5 ± 2.5	7.5 (25.9)

a Nanometers.

b Number of H-bonds.

c Residues in helical structures. Values in parentheses represent their percentage of the total residues.

d In FEL analysis, the area and energy are given in nm^2^ and kJ/mol, respectively.

e Average values of minimum energy structures.

Because of their tertiary structure, the *R*_g_ values for bacteriocin and granulysin show little variation when solvated with the analyzed water models. However, the remaining peptides show a tendency toward folding, which is generally greater when solvated with the OPC3-B model (Table 4). That behavior can be explained by the fact that the OPC3-B model was developed to reproduce intrinsically disordered structures.^46^ Nevertheless, structural compaction differs across models. Figure 5 depicts the final structures of each of the simulated replicas and the folding pattern in each model. While in the OPC3/LEW and SPC/E models, the peptides fold conserving their helical structures, in the OPC3 and OPC3-B models, they tend to adopt disordered configurations. Even though that performance is in good agreement with experimental evidence indicating that these peptides can adopt disordered conformations in aqueous solutions, where the dielectric constant is high,^28–30^ this does not occur in low-polarity media.^31^ Interestingly, with TIP3P, which has the highest dielectric constant (ϵ = 91.3 at 309.65 K), the helix loss was less dramatic than in the OPC3 and OPC3-B models.

**Figure 5:**
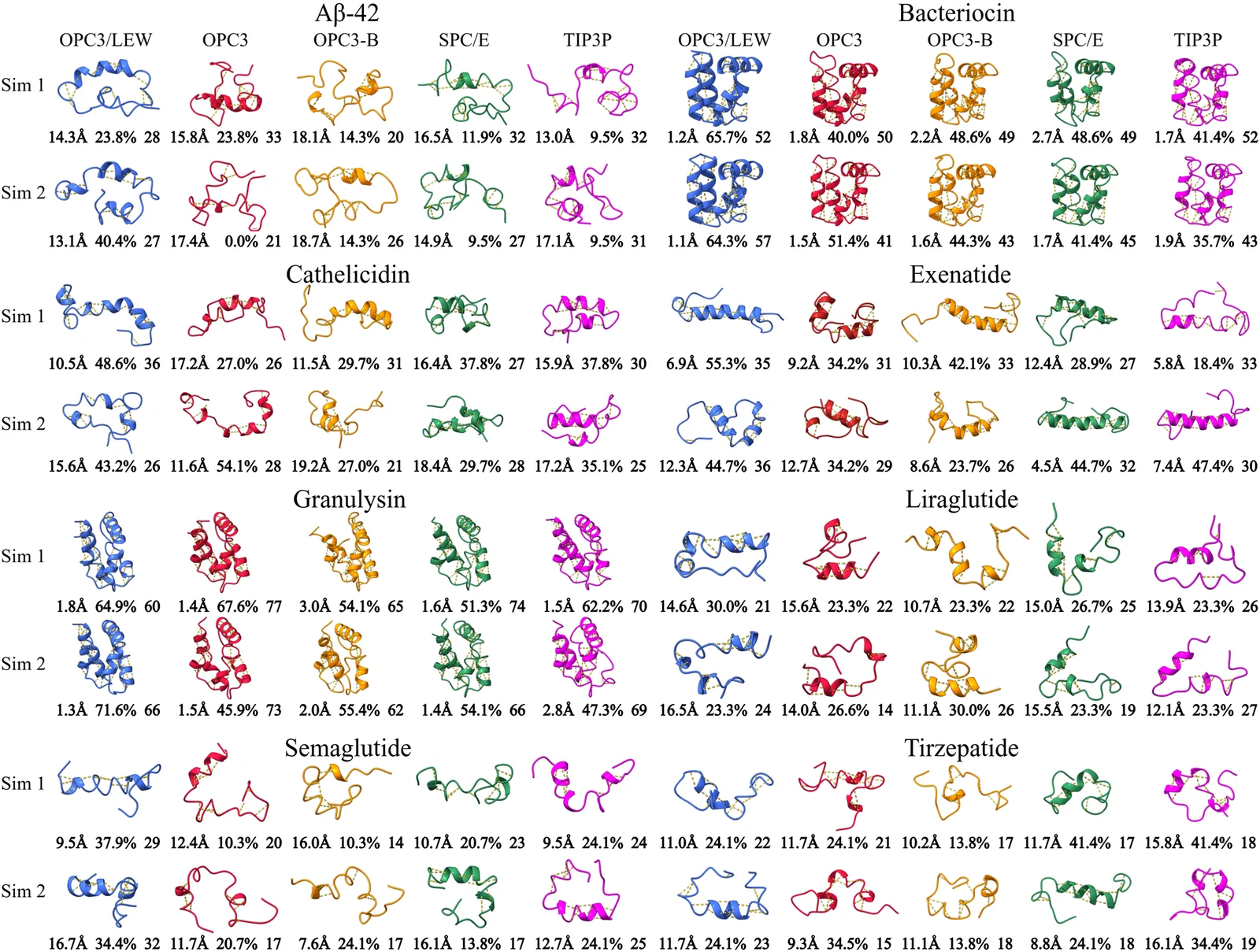
Analysis of the final structures in the MD simulations. The values shown below each structure correspond to the number of H-bonds, the RMSD relative to the experimental structure (nm), and the percentage of residues that form part of helices, respectively. The color code in the structures corresponds to the water model used in the simulation.

In all models, a loss of helical structure was observed, resulting in decreased intramolecular interactions within the peptide chains. According to MD simulations, the OPC3/LEW model shows the greatest conservation/formation of intramolecular H-bonds in the peptides, with an average of 81.6% of these interactions compared to those observed in the experimental structures (Figure 4a). Only for granulysin and liraglutide did the SPC/E and TIP3P models show higher percentages. However, for granulysin, the difference was only 6% relative to the SPC/E model, whereas for liraglutide, the overestimation was 39% relative to its experimental structure. The lowest number of these interactions occurred with tirzepatide (61.3%), although it still exceeded the other models by approximately 13%. Notably, TIP3P ranked second among the models in terms of intramolecular H-bond interaction, averaging 79.1%. The remaining models: SPC/E 74.4%, OPC3 70.6%, and OPC3-B 70.1% (Figure 5 and Table 4).

For the OPC3/LEW model, the increase in intramolecular H-bonds is accompanied by a decrease in peptide-water interactions, due to the low electrostatic environment. Since the structural evolution of peptides depends largely on solvent dynamics, the network of intermolecular H-bonds significantly influences their stability and structure, thereby contributing to their folded state.^12^ In this respect, the design of the new model weakens its competitiveness in solvating peptides and in intermolecular interactions with their polar groups. Compared to the other models, this decrease represents approximately 11% on average.

It is noteworthy that the TIP3P model also exhibits a lower number of interactions with peptides compared to the OPC3, OPC3-B, and SPC/E models (6%). This data appears to contradict the OPC3/LEW results on the correlation between model polarity and H-bond formation. However, when analyzing the interaction parameters of the water models (Table 1), the TIP3P atomic charges are approximately 6% smaller on average. Furthermore, its ϵ_LJ_ parameter, associated with the interaction energy of the oxygen atom, is the second-lowest, only surpassed by OPC3/LEW suggesting that the combination of the nonbonded parameters in the TIP3P model results in a less competitive solvent for hydrogen-bond formation among the analyzed models. This observation can be verified by measuring the self-interaction of TIP3P (HB_wm–wm_, Table 4), which was 8% lower than that of the other models. Since the number of water molecules remained constant in each simulated peptide, the results demonstrate that the TIP3P model tends to form fewer of these types of interactions.

#### The H-Bond Network in the OPC3/LEW Model Determines the Conservation of Helical Structures in the Analyzed Peptides

The performance of the OPC3/LEW model for representing low-electrostatic environments was also evaluated by measuring the percentage of helical conservation in the analyzed peptides. Based on the total number of residues and those forming helices, the percentages were calculated from the final structures obtained in the MD simulations (200 ns; Figure 5). The data exhibit a decrease in the helical structure of peptides across all water models. However, OPC3/LEW preserved the highest percentage of helices, particularly for bacteriocin, exenatide, granulysin, and liraglutide, with decreases of less than 12% relative to the experimental structures (Tables 4 and S5). For their counterparts, the decrease in these structures is similar, with the TIP3P model showing the greatest loss of helical structure. Results also indicate that the number of H-bond_intra_ is not a sufficient parameter to ensure the conservation of secondary structure in peptides and proteins. An example is the TIP3P model, which yielded a higher H-bond_intra_ count than OPC3/LEW in the β-amyloid, granulysin, and liraglutide systems. However, the percentage of helix conservation was lower with TIP3P than in the other models. Upon peptide denaturation, intramolecular interactions with other motifs of its structure are promoted. This tendency toward denaturation is also observed in the other peptides, with tirzepatide being the only peptide that showed more helices with this model.

Undoubtedly, the interaction parameters in the analyzed water models play a crucial role in H-bond formation and, consequently, in the dynamics and plasticity of peptide structures.^12,130^ However, the present analysis demonstrates that the superior performance of the new OPC3/LEW model is related to how its molecules interact with different residues. To clarify this point, the percentages of intra- and intermolecular H-bond occupancy were calculated. Occupancy measures the temporal stability of hydrogen bonds along MD trajectories, thereby indicating whether a protein can lose secondary structure.^130^ Intramolecularly, the results show that OPC3/LEW is not the model with the highest occupancy among the peptides (Figure S6). Average values show that this metric was higher in only three systems: cathelicidin, exenatide, and tirzepatide (Table S6). However, the answer seems to lie in the intermolecular occupancy. The graphs show that although all models exhibit the same interaction pattern with the peptides, occupancy values are systematically lower with OPC3/LEW, decreasing to 16% in semaglutide.

These findings suggest that the hydrogen-bond network in this model minimizes conformational fluctuations compared to those in the other models, thereby improving its ability to preserve helical structures. It is important to note that, as a pure component, OPC3/LEW was the only model that underestimated the experimental value of Δ*H*_vap_. Physically, this property correlates with the energy of the inter- and intramolecular H-bonds that the model can form. Data regarding peptide-water H-bond interactions and their occupancy indicate that OPC3/LEW is less aggressive in competing for the intramolecular H-bonds of peptides, thereby allowing for greater preservation of their helical structures. This can be corroborated by correlating occupancy with helical structure preservation (Figure S7).

#### FEL Analysis Shows Less Flexible and More Stable Configurational Spaces with the OPC3/LEW Model

To validate these results, it was decided to repeat the calculations for minimum-energy structures. For this purpose, a free energy landscape (FEL) analysis was performed using two structural stability indicators as variables: RMSD and radius of gyration. This analysis evaluated the available microstate area (RMSD vs *R*_g_), the global minimum energy of the configurational space, and the conservation of helical structure, as shown in Table 4. The calculations were performed on two replicates of each water model. When several minimum-energy structures were obtained, the reported data are averages. Figure S8 shows the 3D plots of FEL analysis obtained for the β-amyloid peptide. Detailed data for each minimum-energy structure are provided in Table S7.

The FEL area, related to the number of available conformational states,^131,132^ reaches its minimum in systems solvated with OPC3/LEW (Table 4 and Figure S8), indicating fewer transient states and reduced flexibility. These findings are consistent with those observed in systems solvated with the OPC3-B model. As mentioned previously, this model was developed to study intrinsically disordered peptides and proteins, so maximum FEL areas were expected. The data show that the areas with OPC3-B are, on average, 42.3% larger than those with OPC3/LEW, with a maximum value of 78.4% for liraglutide. For the other models, the average of these areas exceeds that of OPC3/LEW by 18.2% (OPC3), 11.5% (SPC/E), and 27.4% (TIP3P).

Regarding free-energy calculations, the new model presents the most stable structures (represented by their energy values at the minima in the FEL maps) for the β-amyloid, cathelicidin, and liraglutide peptides. For bacteriocin, granulysin, and semaglutide, although their energies were not the most favorable, the difference did not exceed 3.3% of the lowest value obtained with the other models. For exenatide, despite an energy difference of 14.1%, it yielded the second-lowest energy. Only for tirzepatide did the difference exceed 18%, making it the most unstable system among those analyzed. These results also correlate with the conservation of secondary structure in the peptides. Upon reaching minimum energy states, configurational transitions involve a higher energy cost, making interactions with the solvent more relevant.^131,132^ According to previous results, these interactions are minimized with the OPC3/LEW model. These results become evident when analyzing the structures in these energy wells. In all models, once the peptide reaches the minimum energy, the difference between the hydrogen-bond values and the conservation of the helical structure is small compared with the structures obtained at the end of the MD pathways. Notably, in the OPC3/LEW model, these structural properties are more likely to increase than decrease.

#### Comparison with Other Low Electrostatic Models

Finally, the performance of OPC3/LEW was assessed by comparing structural metrics with those produced by two other low-electrostatic models: FBA_mem_ and T4F_mem_, here denoted as MEM models. These latter models are flexible; the former has three interaction sites, while the latter has four. Developed by Aguilar-Pineda et al.,^30^ the MEM models revealed the importance of accounting for electrostatic effects in MD simulations for improving the description of structural properties of membrane proteins. Except for β-amyloid, the number of water molecules used to solvate the peptides was the same for the three-site models. For the four-site model, the difference was no greater than 1.6% (Table 5). Comparative analyses of H-bond interactions, intra- and intermolecular occupancy, and FEL were performed using the MD simulations.

**Table 5:** Comparison of Structural Parameters of the Simulated Models^d^

Water	Water	^a^H-bonds				HB occ.			^b^Final struct.			^c^FEL analysis			^b^Min.Energy struct.	
Model	molec	Intra	Pep-wm	wm-wm		Intra	Inter		HBI	Hel_tot_		Area	Energy		HBI	Hel_tot_
Aβ-42		31	-	-		-	-		31	30 (71.4)		-	-		31	30 (71.4)
OPC3/LEW	12542	24 ± 2	91 ± 5	21943 ± 38		85.6	216.7		27.5	14 (33.3)		0.918	16.65		30.0	9.0 (21.4)
FBA_mem_	18166	26 ± 5	100 ± 9	31691 ± 68		116.8	222.5		40.0	3 (7.1)		1.429	18.50		28.0	6.0 (14.3)
T4F_mem_	18960	22 ± 3	99 ± 7	28907 ± 88		85.4	429.3		28.0	0 (0.0)		1.110	11.60		30.0	0.0 (0.0)
Exenatide		33	-	-		-	-		33	21 (53.8)		-	-		33	21 (53.8)
OPC3/LEW	7041	27 ± 2	91 ± 5	12292 ± 28		68.8	228.7		35.5	20 (51.3)		0.296	12.45		37.7	17.7 (46.5)
FBA_mem_	7041	22 ± 3	100 ± 7	11870 ± 43		47.6	244.7		29.0	14 (36.8)		0.923	18.00		38.0	18.0 (47.4)
T4F_mem_	7022	27 ± 4	85 ± 7	10673 ± 54		91.3	409.8		33.0	13 (34.2)		0.183	18.00		39.3	13.7 (35.9)
Liraglutide		14	-	-		-	-		14	11 (35.5)		-	-		14	11 (35.5)
OPC3/LEW	18083	19 ± 2	83 ± 4	31700 ± 46		79.1	254.1		22.5	9 (29.0)		0.567	15.35		21.8	7.5 (25.0)
FBA_mem_	18083	15 ± 2	93 ± 6	30616 ± 68		50.5	277.2		18.0	7 (22.6)		0.634	12.90		16.5	3.5 (11.5)
T4F_mem_	18298	18 ± 3	85 ± 7	27917 ± 88		81.2	499.4		21.0	8 (25.8)		1.152	14.00		22.0	6.0 (20.0)
Semaglutide		36	-	-		-	-		36	26 (86.7)		-	-		36	26 (86.7)
OPC3/LEW	16960	24 ± 2	79 ± 3	29732 ± 42		110.3	256.2		30.5	12 (40.0)		0.720	14.90		25.5	8.0 (27.6)
FBA_mem_	16960	19 ± 3	95 ± 6	28704 ± 63		93.9	295.5		26.0	4 (13.3)		0.970	13.80		23.5	5.5 (19.0)
T4F_mem_	17124	21 ± 3	84 ± 7	26103 ± 91		118.0	520.6		25.0	8 (26.7)		0.760	12.00		31.3	5.8 (19.8)
Tirzepatide		31	-	-		-	-		31	27 (93.1)		-	-		31	27 (93.1)
OPC3/LEW	13886	19 ± 2	65 ± 3	24334 ± 42		64.1	227.5		22.5	8 (27.6)		0.590	12.95		25.5	9.0 (31.0)
FBA_mem_	13886	14 ± 2	79 ± 6	23496 ± 60		50.9	251.3		12.0	9 (31.0)		0.970	11.20		17.0	7.0 (24.1)
T4F_mem_	13668	19 ± 4	61 ± 8	20859 ± 75		90.7	380.5		21.0	5 (17.2)		0.744	16.80		26.0	7.0 (24.1)

a Average number of H-bonds.

b The values indicate the total number of residues that comprise the secondary structure, while the values in parentheses represent the percentages over the total residues of the protein structure.

c Regarding their PDB-ID structures. ^c^In FEL analysis, the area and energy are given in nm^2^ and kJ/mol, respectively.

d All values were obtained as averages over two MD simulations, the original and its replica.

Analysis of H-bonds shows similar behavior between the OPC3/LEW and T4F_mem_ models. In both descriptions the intramolecular interactions within the peptides, mediated by their reduced interaction with the solvent, increase. Despite following the same trend and the values falling within the statistical error, the strong interactions produced by the FBA_mem_ model with the peptides influence the conservation/formation of H-bond_intra_. Additionally, there is a key difference in OPC3/LEW with respect to FBA_mem_ and T4F_mem_, namely that the interactions between water molecules are similar to those in conventional models, thereby preserving their bulk-solvent dynamics.

Regarding H-bond_intra_ occupancy, the FBA_mem_ model again exhibits lower conservation than the other two models. In contrast, T4F_mem_ shows very high values for both intramolecular and intermolecular H-bond occupancy, with the latter being almost twice that observed for OPC3/LEW. However, analysis of H-bond_intra_ interactions and secondary structure in the final configurations again shows that the strength of the OPC3/LEW model in helix conservation lies in the reduction of peptide-water interactions. In this sense, although T4F_mem_ preserves intramolecular bonds for a longer period, its high occupancy of intermolecular interactions allows water molecules to penetrate the peptide matrix, thereby disrupting H-bond_intra_ interactions and decreasing the helix percentage.

As previously discussed, unfolding in peptides leads to increased structural flexibility and a greater number of transient states during simulations. Although all three models exhibit a similar number of H-bond_intra_, the MEM models show that the structures remain compact due to interactions between different motifs within the peptides (Figure 6). The FEL analysis data reveal the difference in how the structures are stabilized in these models. While the structures in the minimum states are energetically comparable in all three models, the FEL area is smaller with OPC3/LEW leading to more stable conformations closer to the native configuration. The difference in how these models stabilize the structures is exemplified by the energy wells for β-amyloid. Table 5 shows that the number of intramolecular H-bonds is the same for the OPC3/LEW and T4F_mem_ models. However, in the latter, the helical structure is not preserved, and β-sheet formation is observed. In contrast, with OPC3/LEW, the defined helical structures are observed in both replicates. The enhanced performance of the OPC3/LEW is attributed to its inclusion of blood plasma surface tension as a target property. This property depends largely on the parametrization of the ϵ_LJ_ value, which is about 30% lower in the OPC3/LEW model than in the MEM models. Because the interaction between water oxygen and peptide atoms is lower in energy, it is unable to compete with the intramolecular H-bond, thereby preserving the helical structure in peptides.

**Figure 6:**
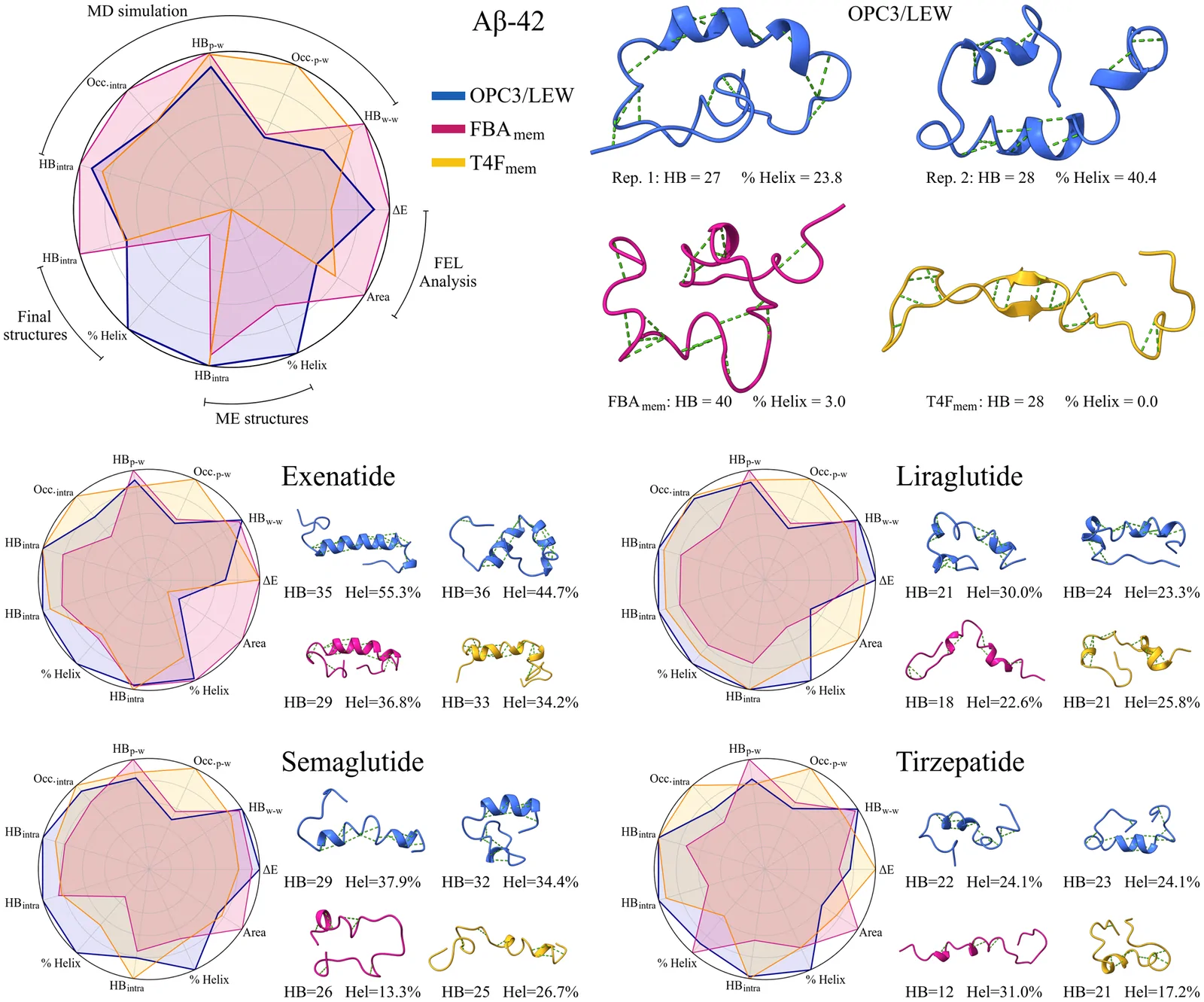
Comparison of structural parameters obtained with the OPC3/LEW model and those obtained with the MEM models FBA_mem_ and T4F_mem_. Only five peptides were evaluated, and the data were obtained as reported by Aguilar-Pineda et al. The values in the radar maps are normalized to the maximum value of each property. The structures correspond to the last frame of each simulation. In both the structures and the series, the same color code was used to represent each water model. Adapted from ref.^30^ Copyright 2025 American Chemical Society.

These results help to clarify why the OPC3/LEW model outperforms the FBA_mem_ and T4F_mem_ models. It is important to note that in the work presented by Aguilar-Pineda et al.,^30^ the best results were obtained with the four-site model, which improved the structural representation of the original model. These findings suggest that this new model can be used in systems where the interface interactions diminish electrostatic effects. Biomolecules such as membrane proteins, whose secondary structure preservation is crucial to their biological function, could benefit from its use as a solvating medium.^30^ Subnanometer channels^133^ and surfaces subjected to electric fields^134^ are further examples. However, the results of the present work indicate that there is still room for further improvement in water models designed for studying such systems.

## Summary and Conclusions

One of the main goals of computational simulations is to closely reproduce the environmental conditions in which biomolecules perform their physiological functions. However, the choice of water model is often based more on computational cost than on its effect on the molecular environment, as is the case with membrane proteins. In these systems, structural, energetic, and interaction properties occur in low-polarity surroundings, environments that the most common and currently available water models do not accurately reproduce.

In order to address this challenge, we present the OPC3/LEW three-site flexible water model, developed to represent low-electrostatic environments in molecular dynamics simulations. Based on the OPC3 model and varying the HOH angle, its interaction parameters were obtained by setting three target properties: a low dielectric constant (20), bulk density (993.7 kg/m^3^), and blood plasma surface tension (52.8 mN/cm^2^), all at physiological temperature and 1 bar of pressure.

The results demonstrate the strengths of this model. As a pure component, OPC3/LEW improves the description of bulk-water properties such as density, diffusion, *r*_O–O_, and *k*_T_ compared to the OPC3-B, SPC/E, and TIP3P models. Notably, OPC3/LEW is the only model that underestimates Δ*H*_vap_, reducing intermolecular hydrogen-bonding interactions. Although this behavior better characterizes low-polarity environments, it increases system fluctuations. Consequently, *C*_P_ and α_P_ are overestimated; therefore, the model is not recommended for systems where these properties are critical. As a solvent for helical peptides, OPC3/LEW promotes the formation of intramolecular bonds within the peptides by reducing their hydrogen bond interactions. These characteristics result in greater preservation of the helical structures characteristic of membrane proteins. Furthermore, the energy analysis of the configurational space shows fewer substates and lower-energy structures when this model is used as a solvent, which could aid in localizing native structures in these environments. Clearly, the main weakness of the OPC3/LEW model lies in its overestimation of the H–O–H angle, which results in an open structure that is rarely addressed in this type of study. However, this methodology preserves the atomic charges of the original model, thereby minimizing the impact on the electrostatic interactions between water molecules and biomolecules.

## Supplementary Material



## Notes

Effect of the opening of the HOH angle on the energy and atomic charges of oxygen in the water molecule (Figure S1); effect of temperature on the tetrahedral structure parameters of water (Figure S2); wheel projections of helical structures of the analyzed peptides (Figure S3); analysis of the helical peptides used to validate the OPC3/LEW water model (Figure S4); analysis of the helical peptides used to validate the OPC3/LEW water model (Figure S5); values of H-bond occupancy per residue of the studied systems (Figure S6); correlation plot of occupancy versus helical structure conservation (Figure S7); free energy landscape (FEL) analysis for the β-amyloid peptide (Figure S8); analysis of FMOs in water molecules (Table S1); tetrahedral order parameters *S*_g_ and *S*_k_, and the coordination numbre *C*_*n*_ for OPC3 water models (Table S2); amphipathic nature of the peptides analyzed; results provided by the AMP Prediction server (Table S3); average stability descriptors of the studied systems (Table S4); comparison of structural parameters of the simulated models (Table S5); average H-bond ocuppancy values (Table S6); measured parameters of minimum energy structures of the studied peptides (Table S7) (PDF)
