# Supplementary material for: OPC3/LEW: A New Flexible Three-Site Water Model to Simulate Low-Electrostatic Environments
Source: ACS Omega. 2026 Oct 21;11(43):67474–92. doi: 10.1021/acsomega.6c08432 (PMC13737924; doi:10.1021/acsomega.6c08432)
Supplement: Supplementary file 1 [file ao6c08432_si_001.pdf]

OPC3/LEW, a new flexible three-site water model to simulate low-electrostatic environments

José Raúl Romero-Figueroa[a], Jorge Alberto Aguilar-Pineda[a], and Minerva González-Melchor[a]*

[a]Instituto de Física "Ing. Luis Rivera Terrazas", Benemérita Universidad Autónoma de Puebla, Av. San Claudio, Edificio IF-1, Ciudad Universitaria, Apdo. Postal J-48, 72570, Puebla, México.

Keywords: Water model; Integral membrane proteins; Blood surface tension Dielectric constant; Electrostatic environment; molecular dynamics

*Authors to whom correspondence should be addressed.
e-mail: minerva@ifuap.buap.mx (M. González-Melchor)

Supplementary Figures

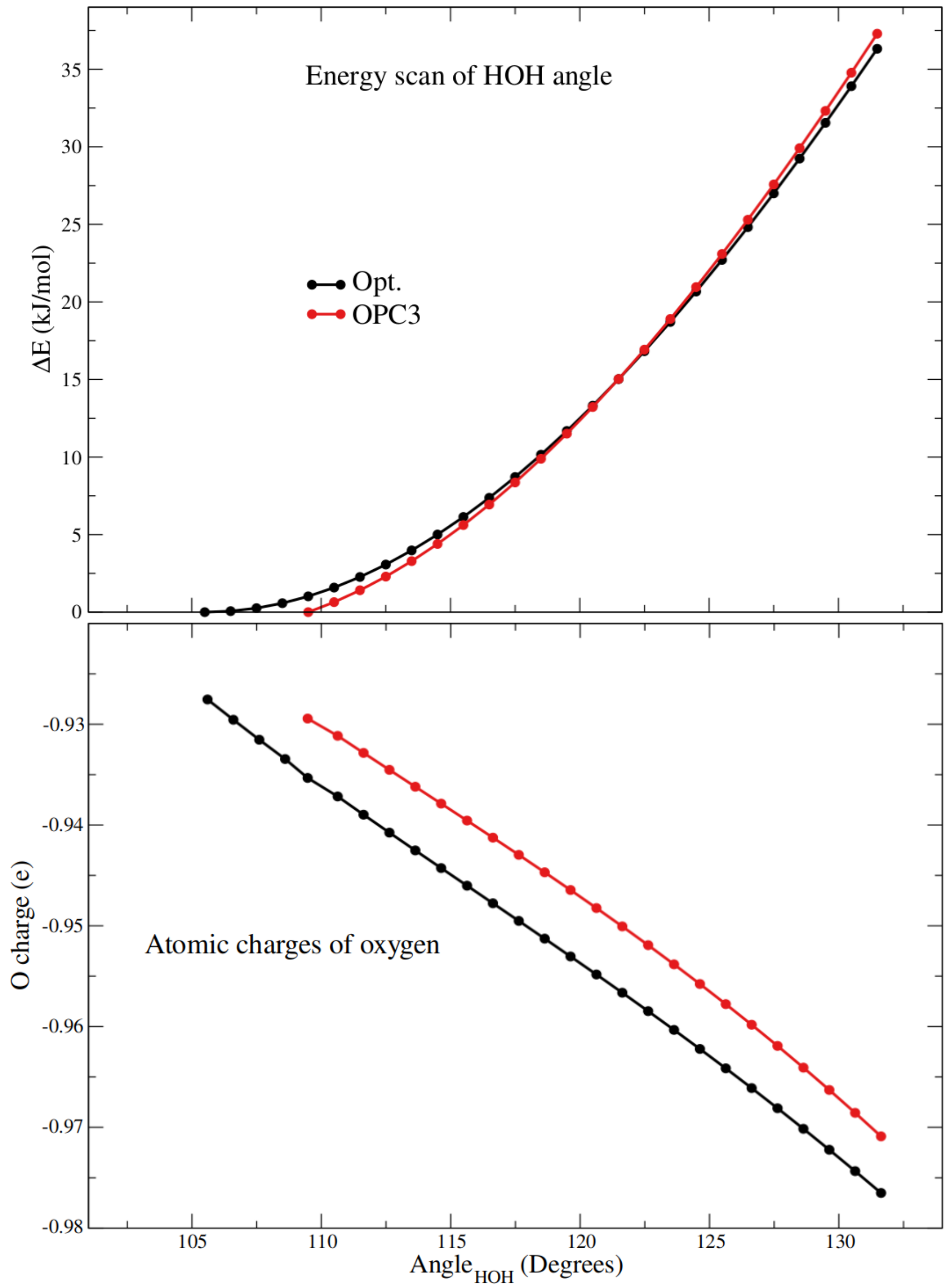

Figure S1. Effect of the opening of the HOH angle on the energy and atomic charges of oxygen in the water molecule.

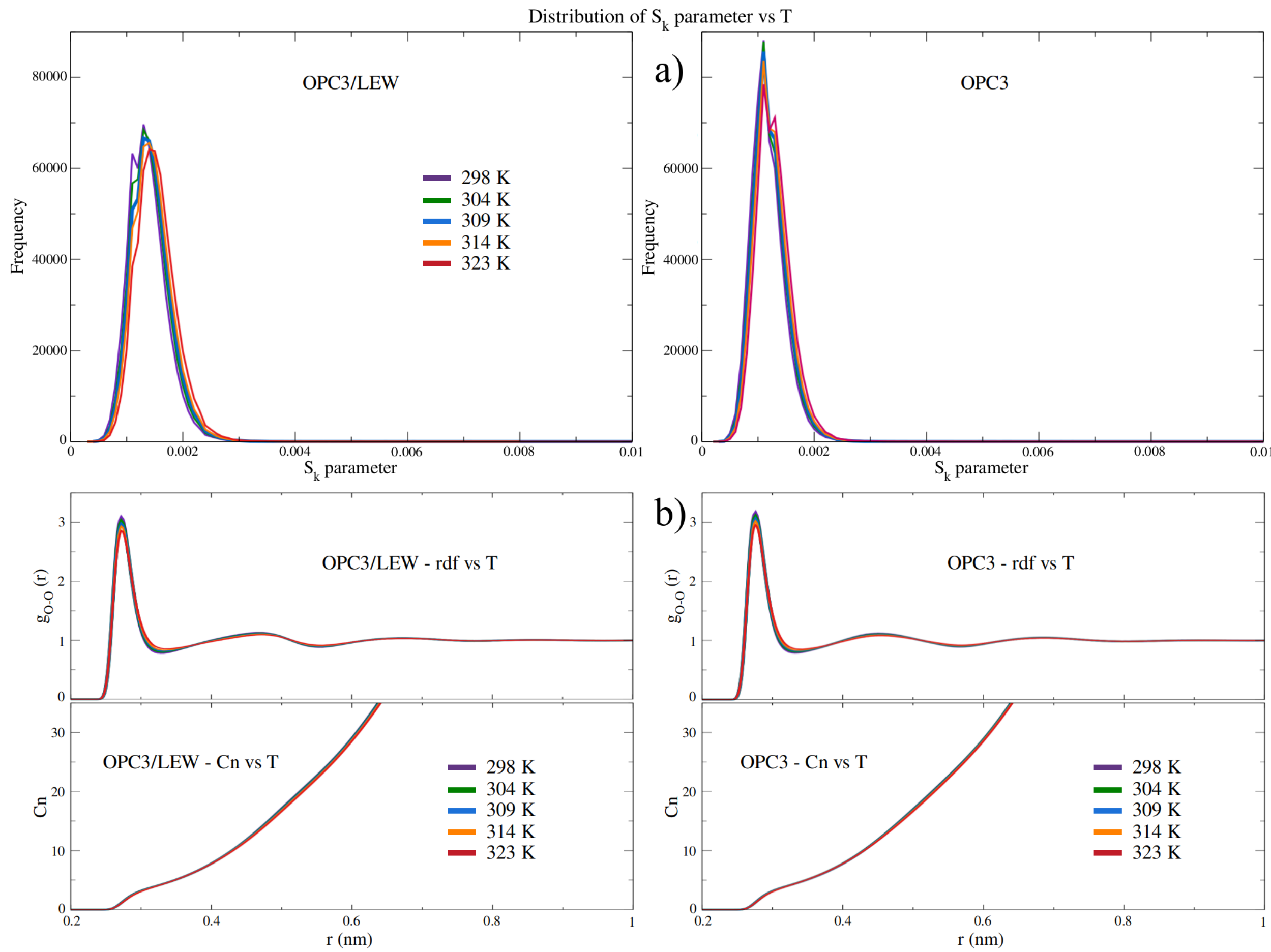

Figure S2. Effect of temperature on the tetrahedral structure parameters of water [1]. Results from the new OPC3/LEW model are shown in the left panel, while those from the OPC3 model [2] are shown in the right panel. a) S_k parameter. b) Oxygen-oxygen radial distribution function and coordination number.

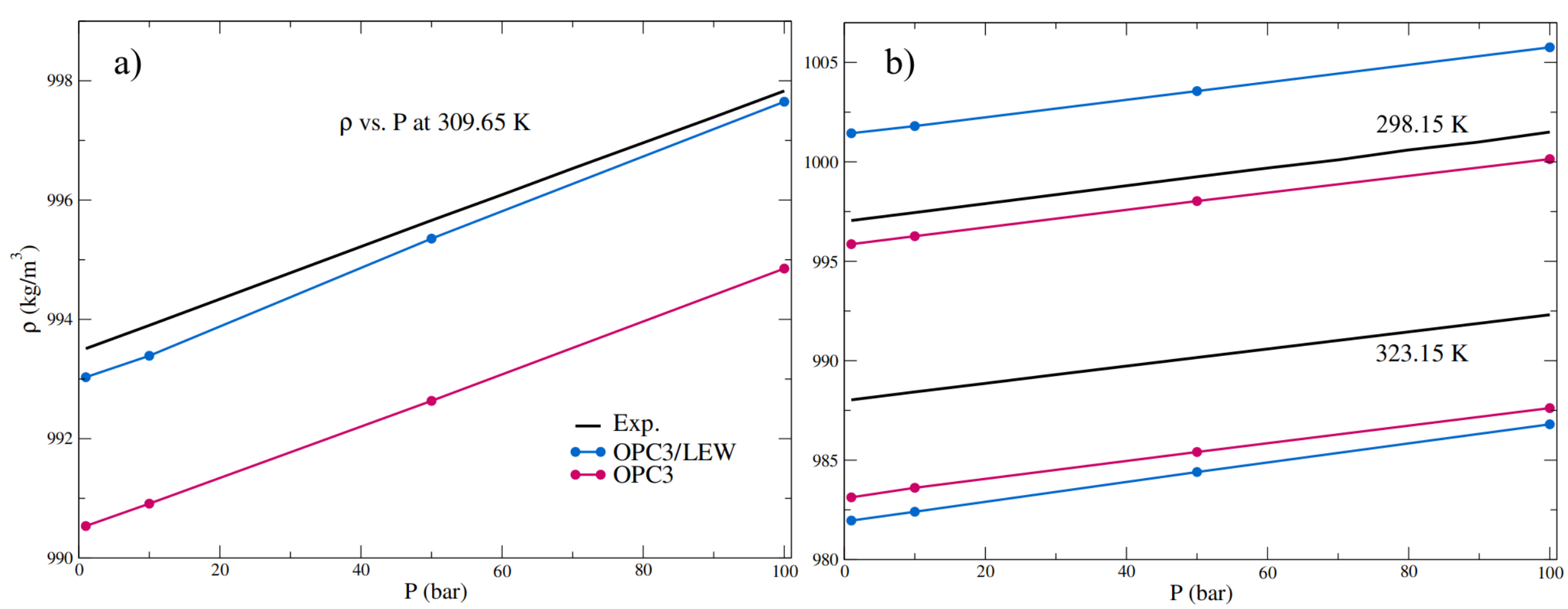

Figure S3. Isothermal density behavior with respect to pressure for the OPC3/LEW (blue line) and OPC3 (red line) models. Experimental data are shown in the black line. a) At 309.65 K, corresponding to the temperature at which the interaction parameters for the OPC3/LEW model were optimized. b) At 298.15 and 323.15 K, the lower and upper limits of the temperature range analyzed.

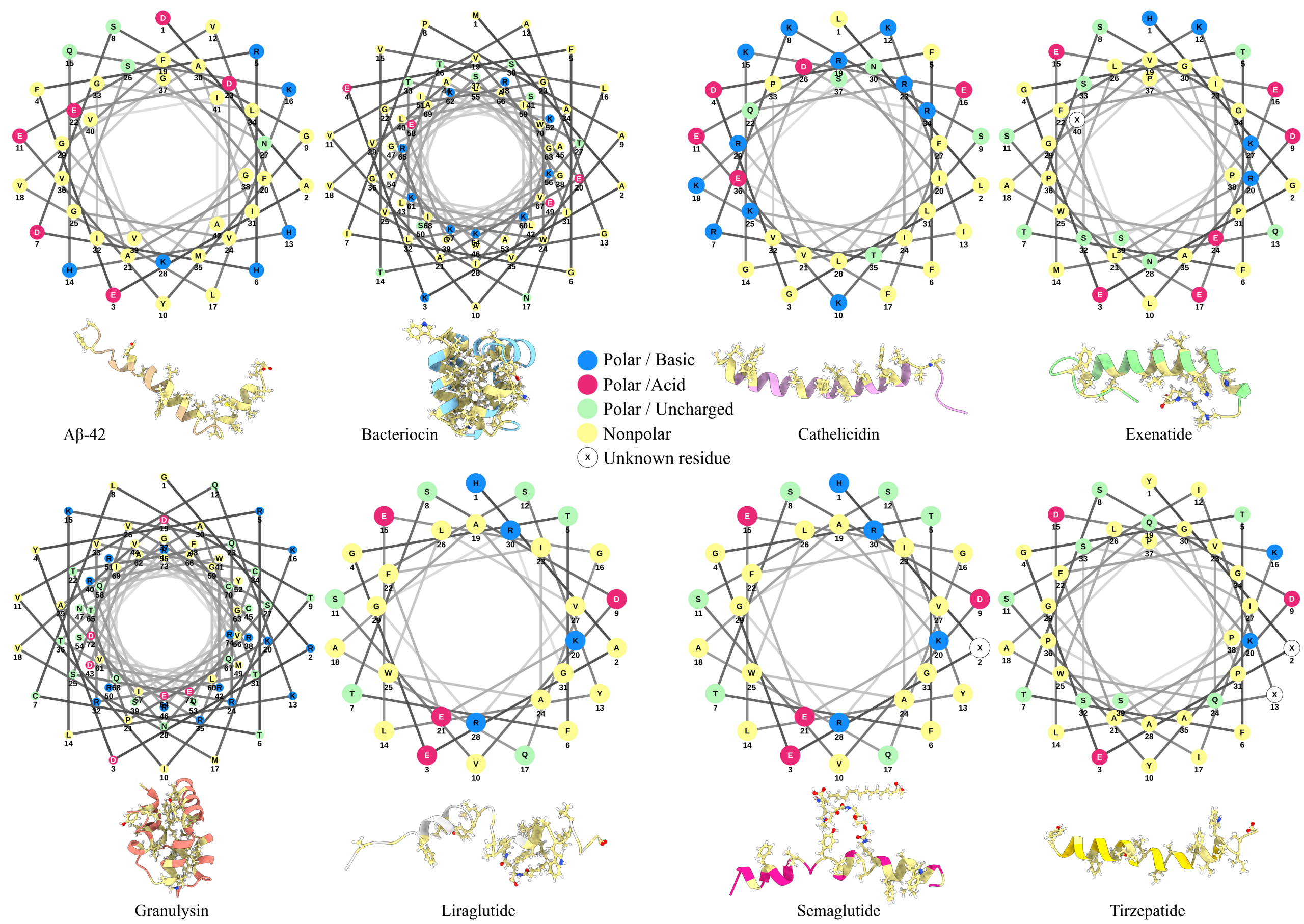

Figure S4. Wheel projections of helical structures of the analyzed peptides. 2D plots were obtained by the NetWheels server [3].

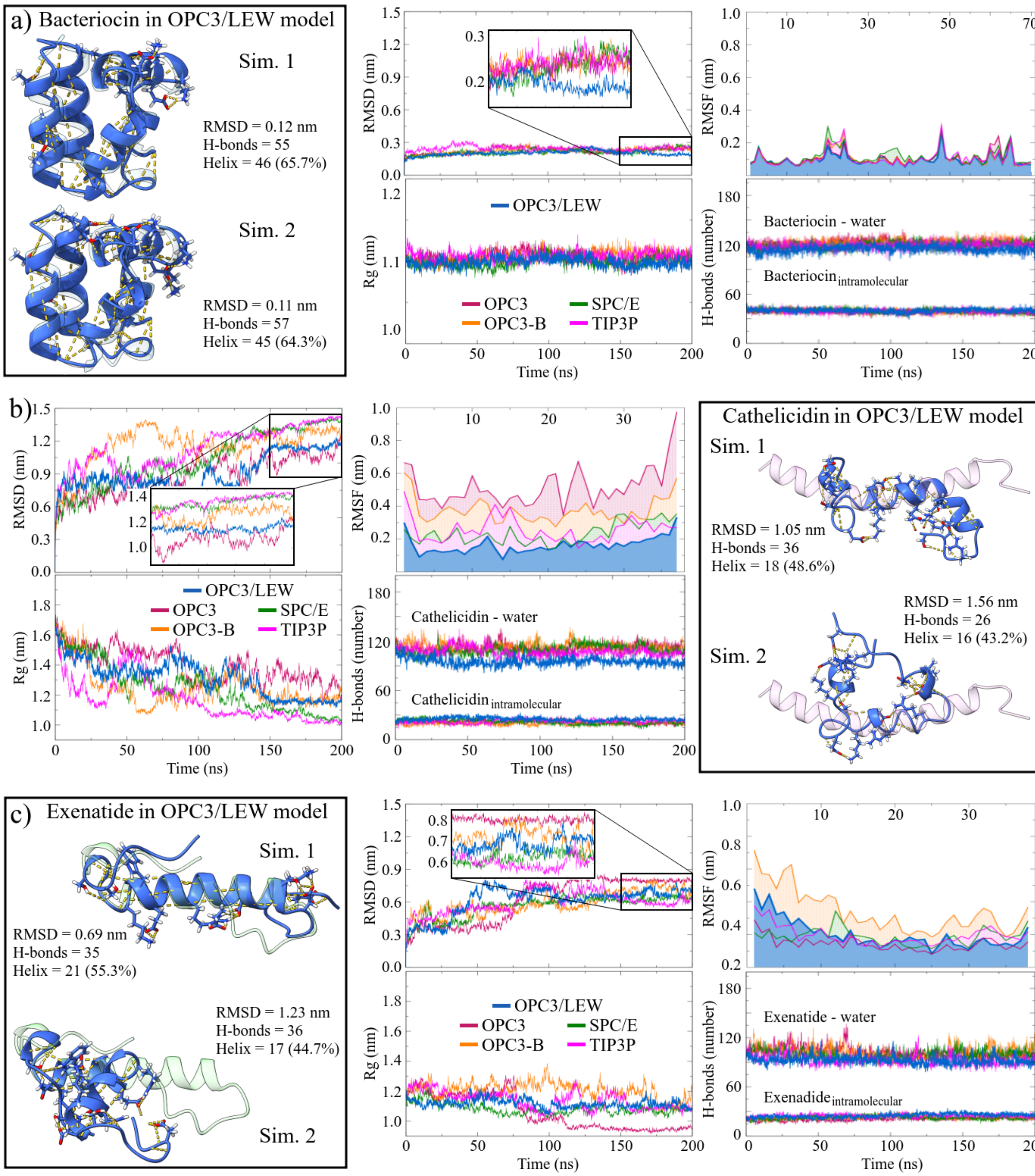

Figure S5. Analysis of the helical peptides used to validate the OPC3/LEW water model. The boxes show structural alignments between the final conformations (original and replicate) from MD simulations and their experimental counterparts. The graphs show stability descriptors for the peptide-water systems: RMSD, radius of gyration, RMSF, and H-bonds. a) bacteriocin (70 residues); b) cathelicidin (37 residues); c) exenatide (38 residues); d) granulysin (74 residues); e) liraglutide (31 residues); f) semaglutide (30 residues); and g) tirzepatide (29 residues). The number of intramolecular hydrogen bonds and the percentage of helical structure are indicated for each peptide.

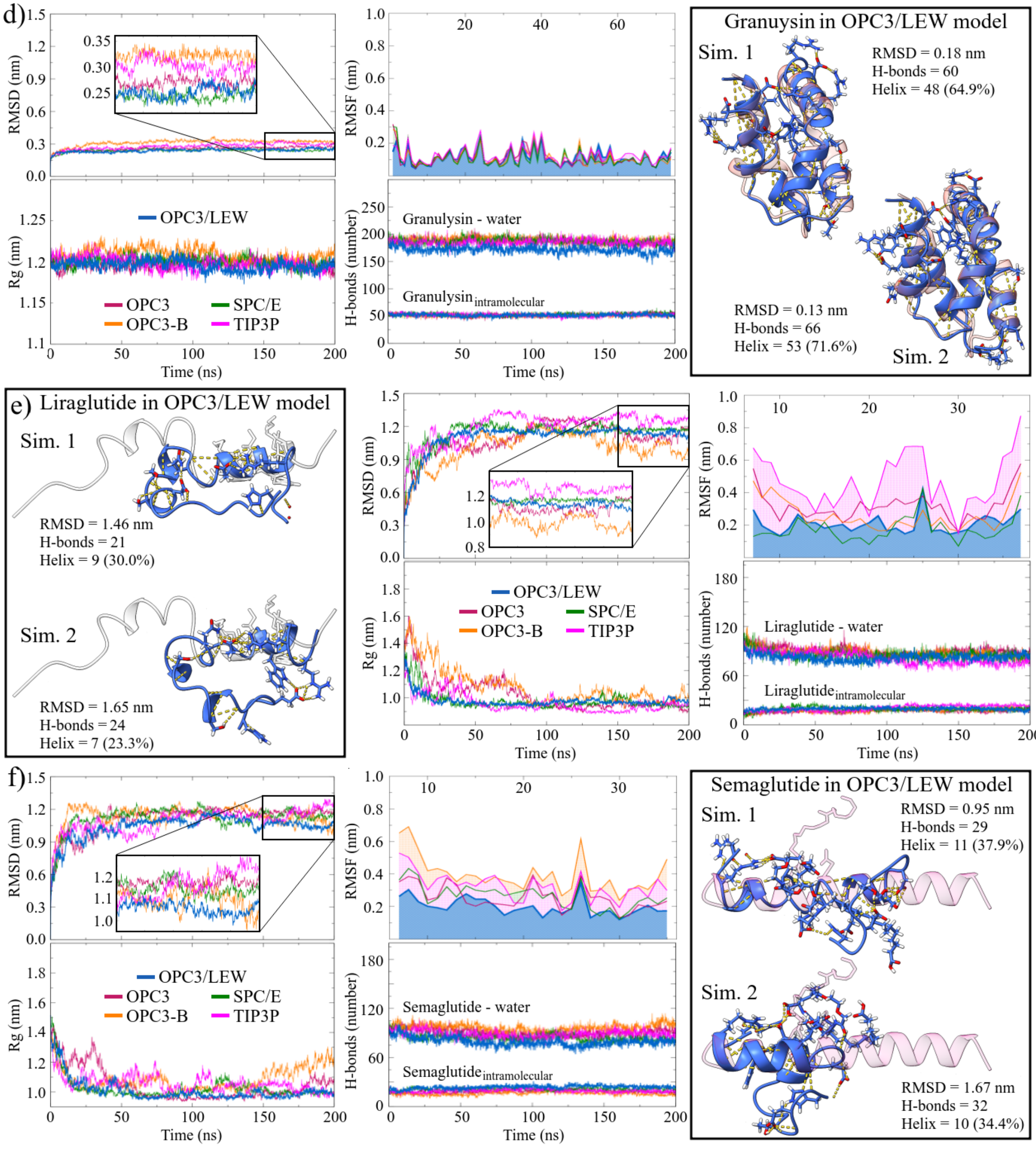

Figure S5-2. Continuation of Figure S5.

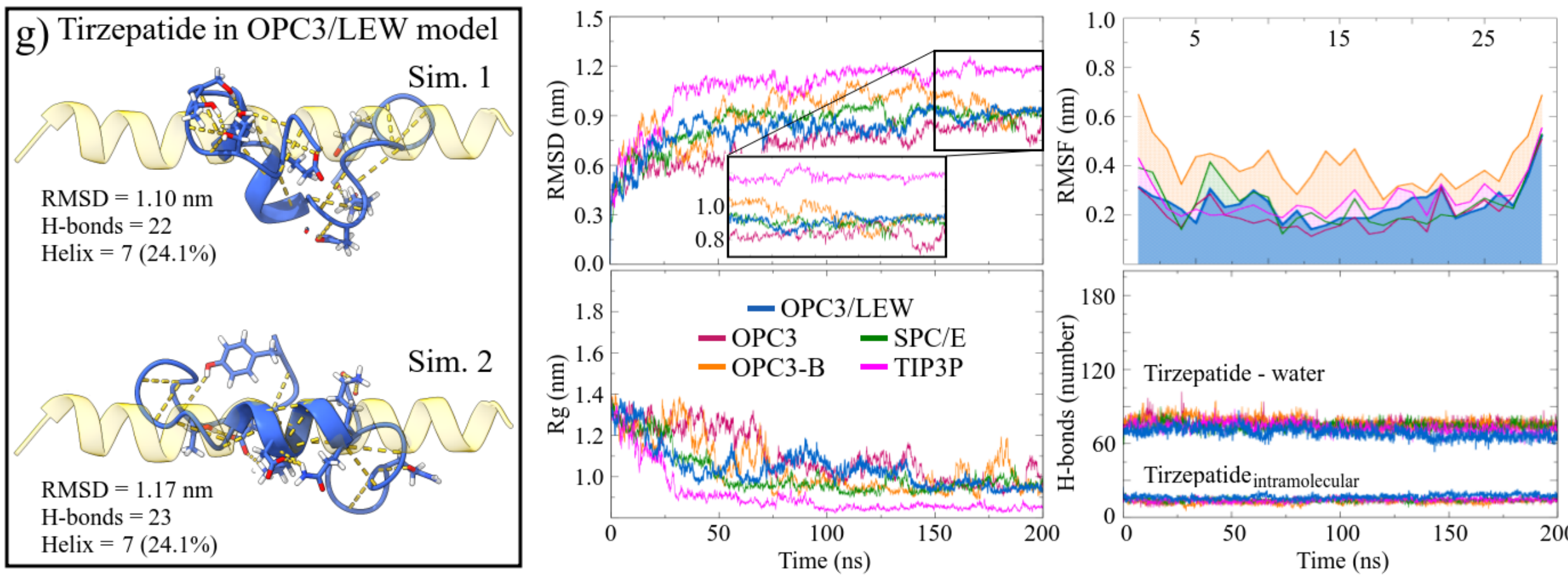

Figure S5-3. Continuation of Figure S5.

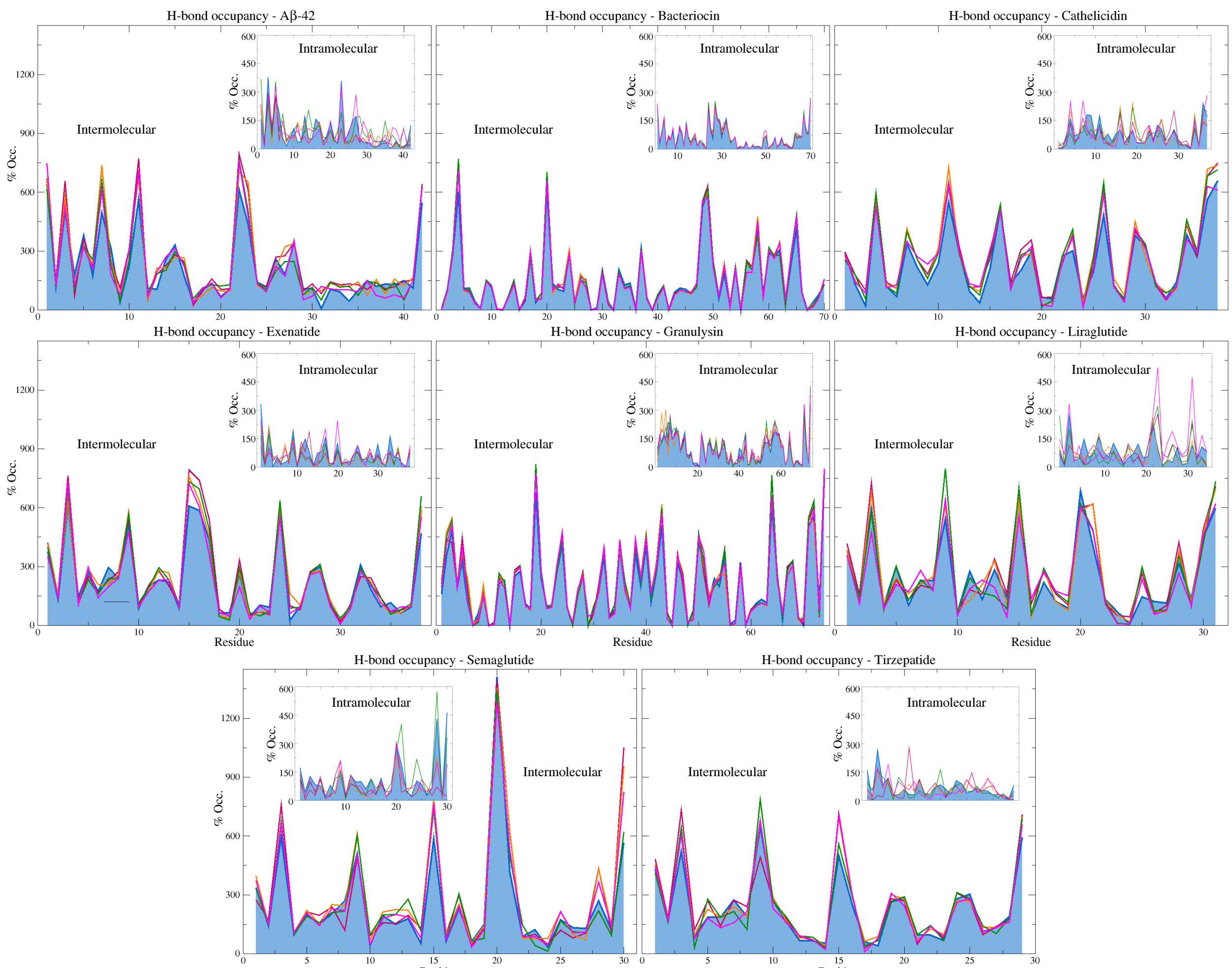

Figure S6. Values of H-bond occupancy per residue of the studied systems. The curve and the blue area represent the OPC3/LEW model's occupancy. For the other models, the same color code was used to represent their H-bond occupancy.

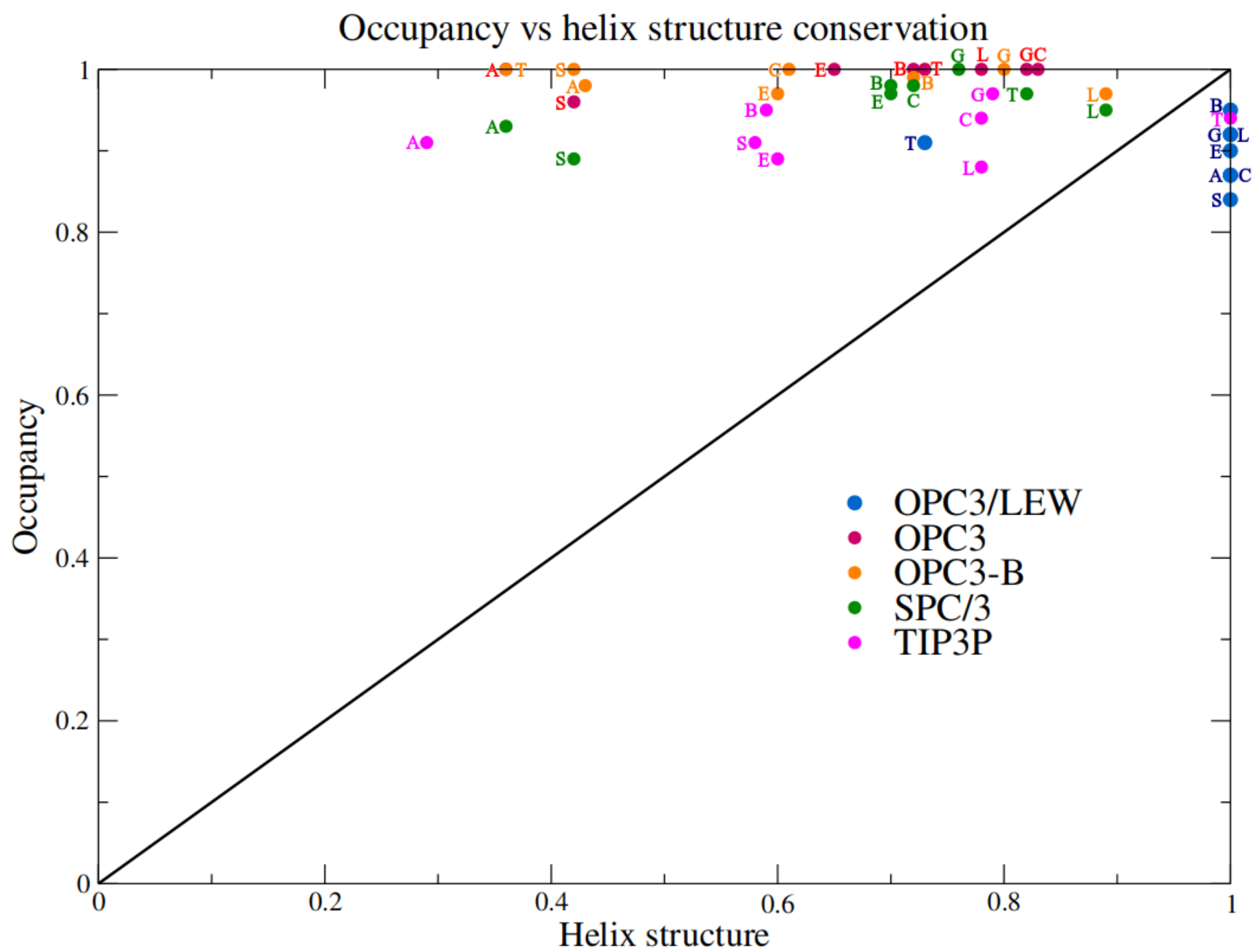

Figure S7. Correlation plot between intermolecular H-bond occupancy and the percentage of conserved helical structure. The values shown were normalized to facilitate visualization. The color coding used for each water model is the same as in the preceding figures. The letters added to each plotted value correspond to the peptide measured: A, Aβ-42; B, bacteriocin; C, cathelicidin; E, exenatide; G, granulysin; L, liraglutide; S, semaglutide; and T, tirzepatide.

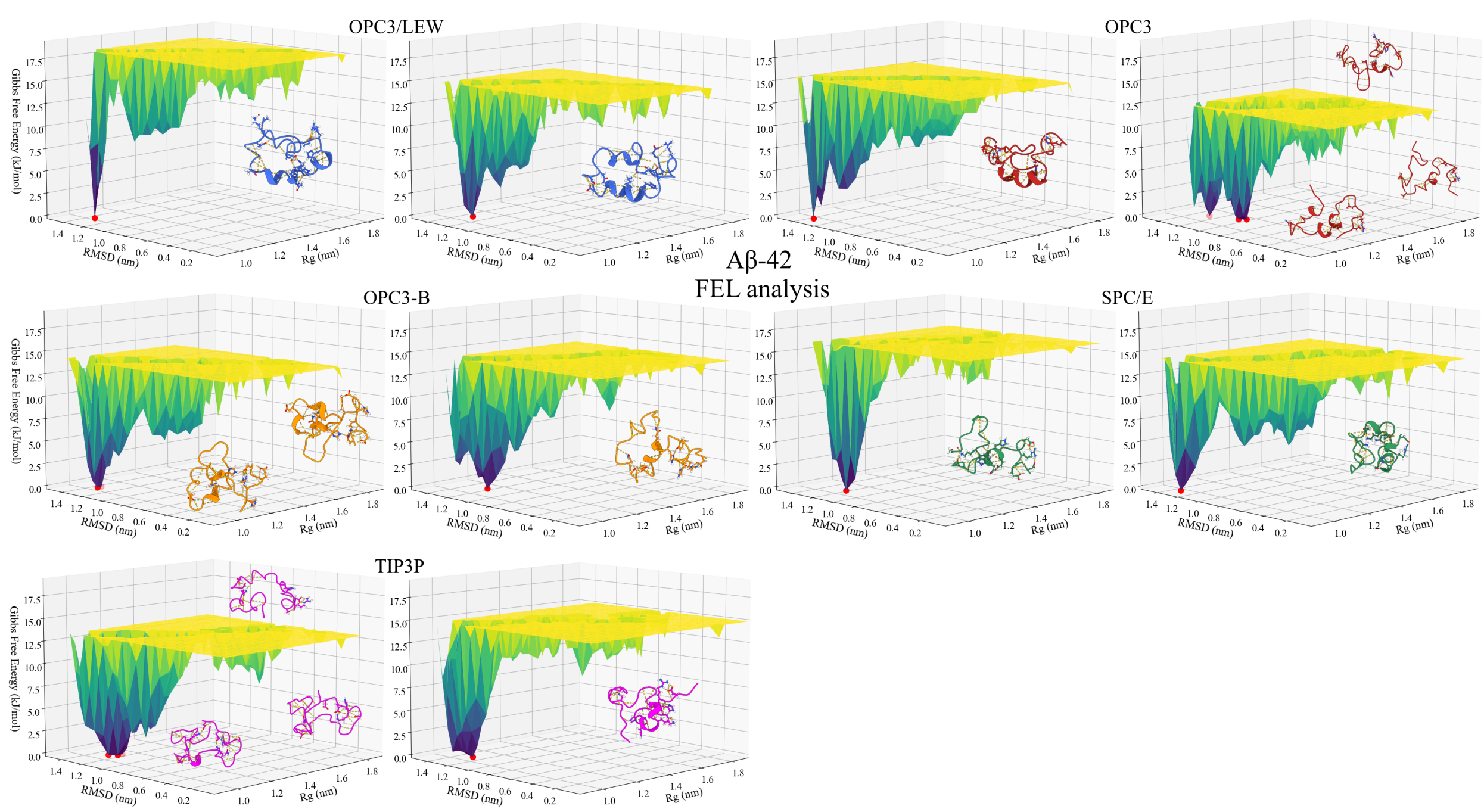

Figure S8. Free energy landscape (FEL) analysis for the β-amyloid peptide. The graphs show the configurational space for this peptide during MD simulations for each water model. For the free energy calculation, two structural variables were chosen: the RMSD and the radius of gyration. The red dots in each graph correspond to the minimum energy structure. The three-dimensional structures at these points are shown in the graphs.

Supplementary Tables

Table S1. Analysis of FMOs in water molecules

Water	FMOs (Hartrees)								
model	$HOMO_{-4}$	$HOMO_{-3}$	$HOMO_{-2}$	$HOMO_{-1}$	HOMO	LUMO	$LUMO_{+1}$	$LUMO_{+2}$	$LUMO_{+3}$
Opt.	-19.16896	-1.02229	-0.54351	-0.39493	-0.31926	0.02489	0.11344	0.15547	0.15557
OPC3/LEW	-19.15939	-1.00519	-0.57716	-0.35259	-0.31397	0.02695	0.10473	0.15143	0.15590
OPC3	-19.16927	-1.01444	-0.54603	-0.38640	-0.31758	0.02413	0.11052	0.15500	0.15558
	ΔE relative to FMOs in the optimized structure (Hartrees)								
OPC3/LEW	-0.00957	-0.01710	0.03365	-0.04234	-0.00529	-0.00206	0.00871	0.00404	-0.00033
OPC3	0.00031	-0.00785	0.00252	-0.00853	-0.00168	0.00076	0.00292	0.00047	-0.00001

Table S2. Tetrahedral order parameters S_g and S_k, and the coordination numbre C_n for OPC3 water models. The C_n values correspond to the distance of the first minimum ($r_{1,min}$) in the radial distribution function plots.

Temperature	(OPC3/LEW)				OPC3			
(K)	S_g	S_k	$r_{1,min}$ (nm)	C_n	S_g	S_k	$r_{1,min}$ (nm)	C_n
298.15	0.094	0.0013	0.328	4.236	0.093	0.0011	0.332	4.395
304.15	0.097	0.0014	0.330	4.373	0.096	0.0011	0.334	4.469
309.65	0.098	0.0013	0.332	4.364	0.097	0.0011	0.336	4.540
314.15	0.100	0.0014	0.332	4.354	0.100	0.0011	0.338	4.617
323.15	0.104	0.0014	0.334	4.412	0.102	0.0011	0.342	4.771

Table S3. Amphipathic nature of the peptides analyzed. Results provided by the AMP Prediction server [4].

Peptide	Amph. Index	Norm. H-M	Linear moment	DCP	PIVA	ASHR	Net charge	Isoelectric point	P-D	Tilt angle
Aβ-42	0.58	0.08	0.30	0.38	1568.84	40.0	-3.0	5.17	26.0	175.0
Bacteriocin	0.83	0.02	0.33	0.32	1317.81	30.0	6.0	10.70	4.0	6.0
Cathelicidin	1.06	0.56	0.20	-0.14	0.00	60.0	6.0	11.15	30.0	50.0
Exenatide	0.66	0.19	0.25	0.10	58.15	30.0	-3.0	4.39	30.0	81.0
Granulysin	0.99	0.23	0.19	-0.14	62.31	30.0	11.0	11.12	30.0	103.0
Liraglutide	0.87	0.07	0.31	0.19	560.93	50.0	-1.0	5.38	18.0	100.0
Semaglutide	0.87	0.07	0.31	0.19	560.93	50.0	-1.0	5.38	18.0	100.0
Tirzepatide	0.72	0.06	0.30	0.26	478.07	50.0	-1.0	4.18	20.0	77.0

Amph: Amphiphilicity; Norm. H-M: Normalized Hydrophobic Moment; DCP: Disordered Conformation Propensity; PIVA: Propensity to in vitro Aggregation; ASRH: Angle Subtended by the Hydrophobic Residues; P-D: Penetration Depth.

Table S4. Average stability descriptors of the studied systems.

Water Model	^{a}RMSD	^{a}RMSF	^{a}Radius of gyration	^{b}SASA	^{c}Volume/ Area	MSD	
						Water	Peptide
Aβ-42			1.63	44.14	1.61		
OPC3/LEW	1.27±0.01	0.16±0.03	1.02±0.01	32.48±0.64	2.06	2.73±0.01	0.02±0.09
OPC3	1.27±0.02	0.20±0.04	1.00±0.02	34.00±0.81	1.98	2.82±0.01	0.14±0.12
OPC3-B	1.32±0.04	0.32±0.07	1.04±0.02	35.27±0.88	1.98	3.64±0.00	0.15±0.05
SPC/E	1.24±0.03	0.24±0.04	1.01±0.01	33.53±0.62	2.02	3.22±0.01	0.16±0.18
TIP3P	1.25±0.07	0.44±0.13	1.02±0.03	35.30±0.88	2.00	6.44±0.08	0.52±0.45
Bacteriocin			1.09	42.85	2.66		
OPC3/LEW	0.19±0.01	0.09±0.04	1.09±0.00	43.94±0.75	2.56	2.65±0.02	0.02±0.11
OPC3	0.24±0.02	0.11±0.05	1.10±0.01	44.79±0.78	2.39	2.77±0.01	0.04±0.04
OPC3-B	0.24±0.02	0.10±0.05	1.11±0.01	45.31±0.68	2.41	3.61±0.03	0.12±0.08
SPC/E	0.23±0.03	0.12±0.05	1.10±0.01	44.59±0.77	2.44	3.16±0.06	0.29±0.19
TIP3P	0.24±0.02	0.11±0.05	1.11±0.01	44.74±0.68	2.51	6.40±0.07	0.20±0.25
Cathalecidin			1.66	46.58	1.54		
OPC3/LEW	1.15±0.02	0.16±0.05	1.15±0.01	36.57±0.63	1.83	2.75±0.03	0.16±0.11
OPC3	1.07±0.07	0.52±0.12	1.30±0.06	41.68±0.90	1.72	2.85±0.01	0.03±0.03
OPC3-B	1.26±0.06	0.38±0.08	1.17±0.03	40.61±0.83	1.68	3.71±0.01	0.12±0.16
SPC/E	1.35±0.04	0.23±0.06	1.07±0.03	37.55±1.63	1.93	3.27±0.05	0.13±0.12
TIP3P	1.36±0.05	0.26±0.07	1.04±0.03	36.50±1.10	1.86	6.61±0.03	0.52±0.05
Exenatide			1.16	32.17	1.85		
OPC3/LEW	0.69±0.03	0.20±0.09	1.11±0.02	32.49±0.71	1.94	2.63±0.02	0.23±0.22
OPC3	0.80±0.01	0.14±0.04	0.94±0.01	30.06±0.66	2.00	2.74±0.02	0.07±0.13
OPC3-B	0.72±0.04	0.34±0.13	1.16±0.06	34.55±1.08	1.75	3.59±0.07	0.04±0.11
SPC/E	0.63±0.03	0.19±0.05	1.07±0.02	32.56±0.67	1.80	3.09±0.05	0.16±0.21
TIP3P	0.60±0.03	0.19±0.06	1.09±0.02	32.28±0.68	1.86	6.26±0.02	0.18±0.17
Granulysin			1.20	53.19	2.31		
OPC3/LEW	0.25±0.01	0.11±0.04	1.19±0.01	52.52±0.81	2.40	2.63±0.04	0.03±0.09
OPC3	0.27±0.01	0.12±0.06	1.20±0.01	53.50±1.01	2.41	2.77±0.02	0.09±0.11
OPC3-B	0.32±0.01	0.11±0.05	1.20±0.01	54.55±0.85	2.42	3.60±0.01	0.17±0.07
SPC/E	0.24±0.01	0.11±0.05	1.20±0.01	53.44±0.84	2.30	3.11±0.02	0.11±0.13
TIP3P	0.30±0.01	0.13±0.05	1.19±0.02	53.14±0.85	2.32	6.35±0.04	0.21±0.26
Liraglutide			1.38	38.69	1.48		
OPC3/LEW	1.14±0.02	0.21±0.06	0.97±0.01	29.79±1.00	1.88	2.77±0.03	0.22±0.33
OPC3	1.11±0.04	0.32±0.09	0.95±0.02	30.55±1.01	1.94	2.86±0.04	0.16±0.15
OPC3-B	0.99±0.05	0.26±0.09	1.00±0.04	31.20±1.04	1.87	3.74±0.04	0.28±0.28
SPC/E	1.17±0.01	0.17±0.08	0.95±0.02	29.34±1.00	1.95	3.24±0.01	0.05±0.18
TIP3P	1.26±0.04	0.47±0.16	0.93±0.03	27.85±0.83	2.00	6.59±0.03	0.31±0.41
Semaglutide			1.52	40.41	1.68		
OPC3/LEW	1.06±0.03	0.19±0.05	0.99±0.01	30.68±0.80	2.03	2.75±0.01	0.18±0.03
OPC3	1.17±0.02	0.26±0.08	1.03±0.05	32.76±1.17	1.84	2.86±0.02	0.40±0.20
OPC3-B	1.10±0.05	0.39±0.11	1.17±0.05	36.98±1.74	1.62	3.67±0.09	0.11±0.19
SPC/E	1.15±0.03	0.26±0.07	1.00±0.01	30.98±0.64	2.00	3.23±0.02	0.16±0.21
TIP3P	1.17±0.06	0.32±0.09	1.02±0.03	32.32±0.96	1.98	6.53±0.07	0.31±0.26
Tirzepatide			1.34	31.37	1.64		
OPC3/LEW	0.91±0.03	0.25±0.08	0.95±0.01	25.99±0.71	1.93	2.73±0.01	0.14±0.24
OPC3	0.83±0.04	0.21±0.09	0.97±0.04	27.62±0.70	1.83	2.83±0.01	0.05±0.07
OPC3-B	0.95±0.06	0.41±0.11	0.99±0.07	28.20±1.08	1.89	3.71±0.01	0.15±0.31
SPC/E	0.90±0.03	0.25±0.10	0.97±0.02	27.10±0.67	1.89	3.23±0.03	0.57±0.18
TIP3P	1.18±0.02	0.27±0.08	0.85±0.01	25.84±0.67	1.91	6.50±0.09	0.54±0.37

[a] Values in nanometers. [b] Values in square nanometers. [c] Values in cubic nanometers/square nanometers. All values were obtained from the last 200 ns of the MD trajectories.

Table S5. Comparison of structural parameters of the simulated models.

Water	Radius of	[a]H-bonds			[b]Secundary structure summary (%)				
Model	gyration (nm)	Intra	Pep-wm	wm-wm	β-strand	α-helix	3_{10} helix	*Total helix*	Other
Aβ-42	1.63	31	-	-	0 (0.0)	30 (71.4)	0 (0.0)	30 (71.4)	12 (28.6)
OPC3/LEW	1.02±0.01	24±2	91±5	21943±38	0 (0.0)	6 (14.3)	8 (19.0)	14 (33.3)	28 (66.7)
OPC3	1.00±0.02	20±2	104±4	22096±34	0 (0.0)	5 (11.9)	0 (0.0)	5 (11.9)	37 (88.1)
OPC3-B	1.04±0.02	19±2	111±4	21574±37	0 (0.0)	3 (7.1)	3 (7.1)	6 (14.3)	36 (85.7)
SPC/E	1.01±0.01	22±2	101±4	21952±35	0 (0.0)	5 (11.9)	0 (0.0)	5 (11.9)	37 (89.1)
TIP3P	1.02±0.03	23±2	99±4	20339±44	2 (4.7)	4 (9.5)	0 (0.0)	4 (9.5)	36 (85.8)
Bacteriocin	1.09	64	-	-	0 (0.0)	54 (77.1)	0 (0.0)	54 (77.1)	16 (22.9)
OPC3/LEW	1.09±0.00	40±3	113±3	21608±37	0 (0.0)	46 (65.7)	0 (0.0)	46 (65.7)	24 (32.3)
OPC3	1.10±0.01	39±2	121±4	21752±36	0 (0.0)	31 (44.3)	2 (2.8)	33 (47.1)	37 (52.9)
OPC3-B	1.11±0.01	38±2	124±4	21239±38	0 (0.0)	29 (41.4)	4 (5.8)	33 (47.1)	37 (52.9)
SPC/E	1.10±0.01	38±2	122±4	21610±37	0 (0.0)	32 (45.7)	0 (0.0)	32 (45.7)	38 (54.3)
TIP3P	1.11±0.01	38±3	120±4	20024±43	0 (0.0)	23 (32.9)	4 (5.7)	27 (38.6)	43 (61.4)
Cathalecidin	1.66	29	-	-	0 (0.0)	30 (81.1)	0 (0.0)	30 (81.1)	7 (18.9)
OPC3/LEW	1.15±0.01	25±2	94±4	41377±52	0 (0.0)	12 (32.4)	6 (16.2)	18 (48.6)	19 (51.4)
OPC3	1.30±0.06	20±2	114±4	41658±49	0 (0.0)	2 (5.4)	13 (35.1)	15 (40.5)	22 (59.5)
OPC3-B	1.17±0.03	21±2	112±4	40677±52	0 (0.0)	11 (29.7)	0 (0.0)	11 (29.7)	26 (70.3)
SPC/E	1.07±0.03	20±2	110±5	41386±49	0 (0.0)	10 (27.0)	3 (8.1)	13 (35.1)	24 (64.9)
TIP3P	1.04±0.03	23±2	105±5	38350±60	0 (0.0)	11 (29.7)	3 (8.1)	14 (37.8)	23 (62.2)
Exenatide	1.16	33	-	-	0 (0.0)	18 (46.2)	3 (7.6)	21 (53.8)	18 (46.2)
OPC3/LEW	1.11±0.02	27±2	91±5	12292±28	0 (0.0)	16 (41.0)	4 (10.3)	20 (51.3)	19 (48.7)
OPC3	0.94±0.01	23±2	98±4	12379±28	0 (0.0)	13 (33.3)	0 (0.0)	13 (33.3)	26 (66.7)
OPC3-B	1.16±0.06	22±2	104±7	12083±29	0 (0.0)	12 (30.8)	0 (0.0)	12 (30.8)	27 (69.2)
SPC/E	1.07±0.02	21±2	101±5	12295±27	0 (0.0)	14 (35.9)	0 (0.0)	14 (35.9)	25 (64.1)
TIP3P	1.09±0.02	25±2	90±4	11393±32	0 (0.0)	12 (30.8)	0 (0.0)	12 (30.8)	27 (69.2)
Granulysin	1.20	64	-	-	0 (0.0)	54 (73.0)	4 (5.4)	58 (78.4)	16 (21.6)
OPC3/LEW	1.19±0.01	52±3	168±7	22883±39	0 (0.0)	48 (64.9)	3 (4.0)	51 (68.9)	23 (31.1)
OPC3	1.20±0.01	54±3	184±5	23027±38	0 (0.0)	40 (54.1)	2 (2.7)	42 (56.8)	32 (43.2)
OPC3-B	1.20±0.01	52±3	188±5	22482±40	0 (0.0)	41 (55.4)	0 (0.0)	41 (55.4)	33 (44.6)
SPC/E	1.20±0.01	55±3	185±5	22877±36	0 (0.0)	37 (50.0)	2 (2.7)	39 (52.7)	35 (47.3)
TIP3P	1.19±0.02	52±3	184±6	21193±46	0 (0.0)	39 (52.7)	1 (1.4)	40 (54.1)	34 (45.9)
Liraglutide	1.38	14	-	-	0 (0.0)	9 (29.0)	2 (6.5)	11 (35.5)	20 (64.5)
OPC3/LEW	0.97±0.01	19±2	83±4	31700±46	0 (0.0)	0 (0.0)	9 (29.0)	9 (29.0)	22 (71.0)
OPC3	0.95±0.02	17±2	90±4	31922±43	0 (0.0)	4 (12.9)	3 (9.7)	7 (22.6)	24 (77.4)
OPC3-B	1.00±0.04	19±2	85±4	31168±46	0 (0.0)	5 (16.1)	3 (9.7)	8 (25.8)	23 (74.2)
SPC/E	0.95±0.02	20±2	84±5	31715±66	0 (0.0)	8 (25.8)	0 (0.0)	8 (25.8)	23 (74.2)
TIP3P	0.93±0.03	23±2	76±4	29385±52	0 (0.0)	0 (0.0)	7 (22.6)	7 (22.6)	24 (77.4)
Semaglutide	1.52	36	-	-	0 (0.0)	26 (86.7)	0 (0.0)	26 (86.7)	4 (13.3)
OPC3/LEW	0.99±0.01	24±2	79±3	29732±42	0 (0.0)	7 (23.3)	5 (16.7)	12 (40.0)	18 (60.0)
OPC3	1.03±0.05	18±2	91±4	29931±40	0 (0.0)	0 (0.0)	5 (16.7)	5 (16.7)	25 (83.3)
OPC3-B	1.17±0.05	15±2	99±5	29221±45	0 (0.0)	0 (0.0)	5 (16.7)	5 (16.7)	25 (83.3)
SPC/E	1.00±0.01	21±2	82±3	29740±43	0 (0.0)	2 (6.7)	3 (10.0)	5 (16.7)	25 (83.3)
TIP3P	1.02±0.03	18±2	90±4	27545±50	0 (0.0)	4 (13.3)	3 (10.0)	7 (23.3)	23 (76.7)
Tirzepatide	1.34	31	-	-	0 (0.0)	27 (93.1)	0 (0.0)	27 (93.1)	2 (6.9)
OPC3/LEW	0.95±0.01	19±2	65±3	24334±42	0 (0.0)	4 (13.8)	4 (13.8)	8 (27.6)	21 (72.4)
OPC3	0.97±0.04	14±1	76±3	24500±38	0 (0.0)	2 (6.9)	6 (20.7)	8 (27.6)	21 (72.4)
OPC3-B	0.99±0.07	13±2	75±3	23922±38	0 (0.0)	4 (13.8)	0 (0.0)	4 (13.8)	25 (86.2)
SPC/E	0.97±0.02	14±2	74±4	24338±39	0 (0.0)	5 (17.2)	4 (13.8)	9 (31.0)	20 (69.0)
TIP3P	0.85±0.01	15±2	70±3	22545±45	0 (0.0)	6 (20.7)	5 (17.2)	11 (37.9)	18 (62.1)

All values were obtained as averages over two MD simulations, the original and its replica. [a]Average number of H-bonds. [b]The values indicate the total number of residues that comprise the secondary structure, while the values in parentheses represent the percentages over the total residues of the protein structure. [c]Regarding their PDB-ID structures.

Table S6. Average H-bond ocuppancy values.

Water	Aβ-42		Bacteriocin		Cathelicidin		Exenatide	
model	Occ.$_{intra}$	Occ.$_{inter}$	Occ.$_{intra}$	Occ.$_{inter}$	Occ.$_{intra}$	Occ.$_{inter}$	Occ.$_{intra}$	Occ.$_{inter}$
OPC3/LEW	85.52	216.66	63.32	146.76	80.21	242.76	68.79	228.65
OPC3	72.70	248.62	64.54	154.46	75.30	279.41	55.91	253.20
OPC3-B	70.62	242.77	62.16	152.81	75.69	278.41	50.54	245.11
SPC/E	100.08	230.53	64.83	150.84	75.41	273.42	47.41	246.58
TIP3P	91.77	226.82	64.08	147.00	75.41	261.47	62.96	225.10

Water	Granulysin		Liraglutide		Semaglutide		Tirzepatide	
model	Occ.$_{intra}$	Occ.$_{inter}$	Occ.$_{intra}$	Occ.$_{inter}$	Occ.$_{intra}$	Occ.$_{inter}$	Occ.$_{intra}$	Occ.$_{inter}$
OPC3/LEW	96.90	206.99	79.05	254.05	110.29	256.22	64.09	227.47
OPC3	95.05	223.26	82.12	277.64	70.21	293.16	55.83	248.96
OPC3-B	93.73	223.51	69.66	268.37	73.82	304.52	57.17	248.26
SPC/E	98.19	223.81	75.44	262.84	116.32	271.20	58.81	242.65
TIP3P	96.51	216.79	112.54	243.30	82.67	278.07	59.41	234.22

Occupancy values were obtained by averaging the percentage occupancy values per residue of each simulated system.

Table S7. Measured parameters of minimum energy structures of the studied peptides.

Water model	^{a}MEF (ns)	RMSD (Å)	HB_{intra}	Helix		3_10		Strand		Rest	
				res	%	res	%	res	%	res	%
**Aβ-42**											
OPC3/LEW - r1	182.6	13.4	30	4	9.5	4	9.5	0	0.0	34	81.0
OPC3/LEW - r2	166.7	13.0	30	10	23.8	0	0.0	0	0.0	32	76.2
OPC3 - r1	194.7	15.7	27	10	23.8	0	0.0	0	0.0	32	76.2
	64.1	12.7	22	8	19.0	3	7.1	0	0.0	31	73.8
OPC3 - r2	113.1	13.8	19	0	0.0	4	9.5	0	0.0	38	90.5
	158.0	17.2	23	0	0.0	4	9.5	0	0.0	38	90.5
OPC3-B - r1	95.0	17.5	24	4	9.5	4	9.5	0	0.0	34	81.0
	115.0	15.4	28	0	0.0	7	16.7	0	0.0	35	83.3
OPC3-B - r2	40.8	13.7	22	4	9.5	0	0.0	0	0.0	38	90.5
SPC/E - r1	78.0	17.1	27	4	9.5	0	0.0	0	0.0	38	90.5
SPC/E - r2	105.9	14.3	26	4	9.5	6	14.3	0	0.0	32	76.2
	155.2	13.6	32	0	0.0	3	7.1	0	0.0	39	92.9
TIP3P - r1	162.2	13.4	29	4	9.5	0	0.0	0	0.0	38	90.5
	187.0	13.1	26	5	11.9	0	0.0	0	0.0	37	88.1
TIP3P - r2	134.3	12.9	26	4	9.5	0	0.0	0	0.0	38	90.5
**Bacteriocin**											
OPC3/LEW - r1	198.9	1.2	50	35	50.0	3	4.3	0	0.0	32	45.7
	114.6	1.0	45	41	58.6	2	2.9	0	0.0	27	38.6
OPC3/LEW - r2	157.0	1.1	53	37	52.9	0	0.0	0	0.0	33	47.1
	189.7	1.1	54	45	64.3	0	0.0	0	0.0	25	35.7
OPC3 - r1	140.2	1.5	46	32	45.7	3	4.3	0	0.0	35	50.0
OPC3 - r2	129.6	1.5	52	32	45.7	2	2.9	0	0.0	36	51.4
OPC3-B - r1	94.0	1.6	48	31	44.3	3	4.3	0	0.0	36	51.4
OPC3-B - r2	70.6	1.9	47	34	48.6	4	5.7	0	0.0	32	45.7
	79.1	2.0	41	29	41.4	0	0.0	0	0.0	41	58.6
SPC/E - r1	67.0	1.2	50	35	50.0	3	4.3	0	0.0	32	45.7
SPC/E - r2	26.3	1.1	56	37	52.9	0	0.0	0	0.0	33	47.1
	41.9	1.0	54	38	54.3	8	11.4	8	11.4	32	45.7
TIP3P - r1	116.0	1.7	43	28	40.0	8	11.4	0	0.0	34	48.6
	129.8	1.6	49	29	41.4	5	7.1	0	0.0	36	51.4
TIP3P - r2	107.6	1.8	42	24	34.3	8	11.4	0	0.0	38	54.3
**Cathelicidin**											
OPC3/LEW - r1	112.6	7.5	37	20	54.1	0	0.0	0	0.0	17	45.9
OPC3/LEW - r2	196.0	15.4	25	8	21.6	6	16.2	0	0.0	23	62.2
OPC3 - r1	82.7	13.9	31	11	29.7	3	8.1	0	0.0	23	62.2
OPC3 - r2	147.0	10.0	29	12	32.4	4	10.8	0	0.0	21	56.8
OPC3-B - r1	155.5	13.7	35	11	29.7	0	0.0	0	0.0	26	70.3
OPC3-B - r2	107.2	15.5	21	8	21.6	7	18.9	0	0.0	22	59.5
	111.7	15.4	17	5	13.5	6	16.2	0	0.0	26	70.3
SPC/E - r1	169.0	15.3	24	9	24.3	0	0.0	0	0.0	28	75.7
SPC/E - r2	177.7	19.3	23	4	10.8	9	24.3	0	0.0	24	64.9
	188.8	16.8	24	4	10.8	9	24.3	0	0.0	24	64.9
TIP3P - r1	29.4	15.3	34	15	40.5	0	0.0	0	0.0	22	59.5
	114.7	17.0	29	7	18.9	3	8.1	0	0.0	27	73.0
TIP3P - r2	180.7	16.9	33	11	29.7	3	8.1	0	0.0	23	62.2

[a]Minimum energy frame.

Table:S7. ...continuation. Measured parameters of minimum energy structures of the studied peptides.

Water model	[a]MEF (ns)	RMSD (Å)	HB_{intra}	Helix		3_10		Strand		Rest	
				res	%	res	%	res	%	res	%
**Exenatide**											
OPC3/LEW - r1	116.5	6.0	41	17	44.7	0	0.0	0	0.0	21	55.3
	118.7	6.2	37	15	39.5	6	15.8	0	0.0	17	44.7
OPC3/LEW - r2	148.4	10.2	35	15	39.5	0	0.0	0	0.0	23	60.5
OPC3 - r1	199.5	9.1	27	12	31.6	0	0.0	0	0.0	26	68.4
OPC3 - r2	146.8	13.2	32	16	42.1	0	0.0	0	0.0	22	57.9
OPC3-B - r1	101.1	7.1	38	16	42.1	0	0.0	0	0.0	22	57.9
OPC3-B - r2	54.0	4.4	30	13	34.2	0	0.0	0	0.0	25	65.8
SPC/E - r1	125.3	10.3	24	8	21.1	0	0.0	0	0.0	30	78.9
	33.7	3.9	32	12	31.6	7	18.4	0	0.0	19	50.0
SPC/E - r2	81.7	4.5	32	19	50.0	0	0.0	0	0.0	19	50.0
	143.6	4.0	35	18	47.4	0	0.0	0	0.0	20	52.6
TIP3P - r1	181.1	5.9	37	9	23.7	6	15.8	0	0.0	23	60.5
TIP3P - r2	151.3	6.7	36	17	44.7	0	0.0	4	10.5	17	44.7
**Granulysin**											
OPC3/LEW - r1	7.5	1.7	69	43	58.1	3	4.1	0	0.0	28	37.8
OPC3/LEW - r2	123.4	1.3	73	39	52.7	6	8.1	0	0.0	29	39.2
	41.0	1.5	65	39	52.7	4	5.4	0	0.0	31	41.9
	49.7	1.4	60	38	51.4	3	4.1	0	0.0	33	44.6
OPC3 - r1	117.3	1.5	67	37	50.0	5	6.8	0	0.0	32	43.2
	190.6	1.2	73	40	54.1	7	9.5	0	0.0	27	36.5
OPC3 - r2	122.9	1.3	74	39	52.7	2	2.7	0	0.0	33	44.6
	144.5	1.3	60	45	60.8	0	0.0	0	0.0	29	39.2
	119.0	2.8	69	40	54.1	0	0.0	0	0.0	34	45.9
OPC3-B - r1	161.1	3.1	71	35	47.3	0	0.0	0	0.0	39	52.7
	196.5	3.2	69	45	60.8	0	0.0	0	0.0	29	39.2
	59.8	1.8	58	39	52.7	0	0.0	0	0.0	35	47.3
OPC3-B - r2	159.4	1.6	63	36	48.6	5	6.8	0	0.0	33	44.6
	191.8	1.6	70	38	51.4	0	0.0	0	0.0	36	48.6
	13.5	1.5	67	42	56.8	0	0.0	0	0.0	32	43.2
SPC/E - r1	31.4	1.5	70	47	63.5	0	0.0	0	0.0	27	36.5
	36.6	1.5	65	44	59.5	0	0.0	0	0.0	30	40.5
	71.8	1.4	72	47	63.5	2	2.7	0	0.0	25	33.8
	35.3	1.3	71	51	68.9	0	0.0	0	0.0	23	31.1
SPC/E - r2	101.6	1.3	61	37	50.0	3	4.1	0	0.0	34	45.9
	165.8	1.4	70	35	47.3	3	4.1	0	0.0	36	48.6
	189.5	1.5	68	38	51.4	0	0.0	0	0.0	36	48.6
TIP3P - r1	122.8	1.4	63	39	52.7	0	0.0	0	0.0	35	47.3
	182.6	1.4	69	44	59.5	0	0.0	0	0.0	30	40.5
TIP3P - r2	107.7	3.0	62	32	43.2	2	2.7	0	0.0	38	51.4

[a]Minimum energy frame.

Table:S7. ...continuation. Measured parameters of minimum energy structures of the studied peptides.

Water model	^{a}MEF (ns)	RMSD (Å)	HB_{intra}	Helix res	Helix %	3_10 res	3_10 %	Strand res	Strand %	Rest res	Rest %
**Liraglutide**											
OPC3/LEW - r1	65.2	13.9	22	4	13.3	3	10.0	0	0.0	23	76.7
	159.9	15.4	17	0	0.0	3	10.0	0	0.0	27	90.0
OPC3/LEW - r2	174.0	15.585	26	4	13.3	6	20.0	0	0.0	20	66.7
	176.0	18.584	22	4	13.3	6	20.0	0	0.0	20	66.7
OPC3 - r1	96.4	16.217	24	4	13.3	6	20.0	0	0.0	20	66.7
	109.9	16.456	19	0	0.0	6	20.0	0	0.0	24	80.0
OPC3 - r2	92.0	16.743	17	0	0.0	9	30.0	0	0.0	21	70.0
OPC3-B - r1	97.7	15.398	19	0	0.0	4	13.3	0	0.0	26	86.7
OPC3-B - r2	195.5	11.05	24	0	0.0	3	10.0	0	0.0	27	90.0
SPC/E - r1	117.2	15.151	23	8	26.7	0	0.0	0	0.0	22	73.3
SPC/E - r2	117.2	17.32	19	5	16.7	0	0.0	0	0.0	25	83.3
TIP3P - r1	87.5	16.921	20	4	13.3	0	0.0	0	0.0	26	86.7
TIP3P - r2	135.0	15.643	26	4	13.3	0	0.0	0	0.0	26	86.7
**Semaglutide**											
OPC3/LEW - r1	81.8	9.1	20	8	27.6	0	0.0	0	0.0	21	72.4
OPC3/LEW - r2	160.2	16.8	31	8	27.6	0	0.0	0	0.0	21	72.4
OPC3 - r1	172.7	11.918	20	6	20.7	0	0.0	0	0.0	23	79.3
OPC3 - r2	76.3	11.03	20	4	13.8	0	0.0	0	0.0	25	86.2
OPC3-B - r1	121.9	11.26	23	4	13.8	3	10.3	0	0.0	22	75.9
OPC3-B - r2	50.5	17.743	21	7	24.1	8	27.6	0	0.0	14	48.3
	50.8	17.897	15	10	34.5	5	17.2	0	0.0	14	48.3
SPC/E - r1	98.2	11.5	22	4	13.8	3	10.3	0	0.0	22	75.9
SPC/E - r2	59.7	16.704	22	4	13.8	3	10.3	0	0.0	22	75.9
	91.1	12.616	25	4	13.8	6	20.7	0	0.0	19	65.5
TIP3P - r1	155.2	7.998	16	4	13.8	0	0.0	0	0.0	25	86.2
	156.6	7.989	21	5	17.2	3	10.3	0	0.0	21	72.4
TIP3P - r2	129.6	12.383	24	4	13.8	4	13.8	0	0.0	21	72.4
**Tirzepatide**											
OPC3/LEW - r1	124.2	9.9	26	7	24.1	3	10.3	0	0.0	19	65.5
OPC3/LEW - r2	158.9	11.6	25	8	27.6	0	0.0	0	0.0	21	72.4
OPC3 - r1	26.1	7.567	19	15	51.7	2	6.9	0	0.0	12	41.4
	84.7	6.061	17	8	27.6	0	0.0	0	0.0	21	72.4
OPC3 - r2	193.7	9.811	15	4	13.8	9	31.0	0	0.0	16	55.2
	196.6	9.383	15	4	13.8	6	20.7	0	0.0	19	65.5
	59.9	13.377	17	8	27.6	2	6.9	0	0.0	19	65.5
OPC3-B - r1	75.0	11.43	20	8	27.6	0	0.0	0	0.0	21	72.4
	133.6	10.505	14	8	27.6	0	0.0	0	0.0	21	72.4
OPC3-B - r2	185.8	11.78	20	8	27.6	0	0.0	0	0.0	21	72.4
	190.3	11.289	19	8	27.6	0	0.0	0	0.0	21	72.4
SPC/E - r1	75.0	11.707	19	9	31.0	3	10.3	0	0.0	17	58.6
	105.8	14.677	15	4	13.8	7	24.1	0	0.0	18	62.1
SPC/E - r2	50.3	10.308	20	7	24.1	0	0.0	0	0.0	22	75.9
	83.7	9.592	15	8	27.6	2	6.9	0	0.0	19	65.5
TIP3P - r1	95.8	12.961	18	6	20.7	0	0.0	0	0.0	23	79.3
TIP3P - r2	194.2	17.891	23	4	13.8	5	17.2	0	0.0	20	69.0

[a]Minimum energy frame.

References

[1] P.-L. Chau, A. Hardwick, A new order parameter for tetrahedral configurations, Molecular Physics 93 (1998) 511–518.

[2] S. Izadi, A. V. Onufriev, Accuracy limit of rigid 3-point water models, The Journal of chemical physics 145 (2016).

[3] A. R. Mól, M. S. Castro, W. Fontes, Netwheels: A web application to create high quality peptide helical wheel and net projections, Journal of Bioinformatics and Systems Biology 7 (2024) 98–100.

[4] B. Vishnepolsky, M. Pirtskhalava, Prediction of linear cationic antimicrobial peptides based on characteristics responsible for their interaction with the membranes, Journal of chemical information and modeling 54 (2014) 1512–1523.
